# A high-quality genome of *Eragrostis curvula* grass provides insights into Poaceae evolution and supports new strategies to enhance forage quality

**DOI:** 10.1038/s41598-019-46610-0

**Published:** 2019-07-15

**Authors:** J. Carballo, B. A. C. M. Santos, D. Zappacosta, I. Garbus, J. P. Selva, C. A. Gallo, A. Díaz, E. Albertini, M. Caccamo, V. Echenique

**Affiliations:** 1 Centro de Recursos Naturales Renovables de la Zona Semiárida (CERZOS – CCT – CONICET Bahía Blanca) and Departamento de Agronomía, Universidad Nacional del Sur, Camino de la Carrindanga km 7, 8000 Bahía Blanca, Argentina; 2 NIAB, Huntingdon Road, Cambridge, CB3 0LE UK; 3 Università degli Studi di Perugia, Dip. di Scienze Agrarie, Alimentari e Ambientali, Borgo XX Giugno 74, 06121 Perugia, Italy

**Keywords:** Agricultural genetics, Agricultural genetics, Polyploidy in plants

## Abstract

The Poaceae constitute a taxon of flowering plants (grasses) that cover almost all Earth’s inhabitable range and comprises some of the genera most commonly used for human and animal nutrition. Many of these crops have been sequenced, like rice, Brachypodium, maize and, more recently, wheat. Some important members are still considered orphan crops, lacking a sequenced genome, but having important traits that make them attractive for sequencing. Among these traits is apomixis, clonal reproduction by seeds, present in some members of the Poaceae like *Eragrostis curvula*. A *de novo*, high-quality genome assembly and annotation for *E*. *curvula* have been obtained by sequencing 602 Mb of a diploid genotype using a strategy that combined long-read length sequencing with chromosome conformation capture. The scaffold N50 for this assembly was 43.41 Mb and the annotation yielded 56,469 genes. The availability of this genome assembly has allowed us to identify regions associated with forage quality and to develop strategies to sequence and assemble the complex tetraploid genotypes which harbor the apomixis control region(s). Understanding and subsequently manipulating the genetic drivers underlying apomixis could revolutionize agriculture.

## Introduction

Climate change modeling predicts sustained elevated temperatures in which C4 grasses will thrive^1^. *E*. *curvula* (Schrad.) Nees (weeping lovegrass) is a C4 perennial grass member of the Poaceae family, Chloridoideae subfamily. The *E*. *curvula* complex has a basic chromosome number of X = 10 and includes cytotypes with different ploidy levels (from 2X to 8X) that may undergo sexual reproduction and facultative or obligate apomixis^2^. Its drought tolerance and capacity to grow in sandy soils make it highly valued, especially for cattle feed in semiarid regions^3^. However, weeping lovegrass, like other C4 species, has lower nutritional quality compared to C3 species. Different molecular strategies have been developed in order to increase forage quality. Recently^4^, the genes for class I and class II caffeoyl shikimate esterase (CSE) have been discovered to be involved in lignin regulation being interesting targets to improve forage quality through genetic engineering^5^. In addition, *E*. *curvula* has been suggested as a potential biofuel crop^6^. In this context, the availability of a high-quality genome assembly for weeping lovegrass is essential to enable genetic improvement that aims to increase its digestibility and energy provision. Moreover, since *E*. *curvula* is a species adapted to high temperature, high radiation and drought, the characterization of the WRKY transcription factors could be central to understand the mechanism involved in resilience in case of environmental stresses.

*Eragrostis* is a poorly studied polyphyletic genus, with more than 400 species^7^, originating from Africa and now distributed in tropical and mid warm-season regions all over the world. *Eragrostis tef*, a cereal from Ethiopia, and *E*. *curvula*, a forage grass from the south of Africa, are the best-studied species of the genus. *Setaria italica* and *Sorghum bicolor* genomes were reported to be the closest relatives of *E*. *tef*^8^. However, comparisons of the *E*. *curvula* transcriptome with the *E*. *tef* genome and transcriptome sequences^9^ support the involvement of *E*. *curvula* in *E*. *tef* evolution. Like *E*. *tef*^10^, *E*. *curvula* is classified as an orphan, or underutilized, crop and despite its importance, very little research emphasis has been given to this species.

Until recently, the major limitation in any genome assembly project was given by the short length of the reads obtained^11^. The advent of new platforms for long molecule sequencing, such as the PacBio Sequel System and Oxford Nanopore Technologies systems, has greatly contributed in overcoming this limitation^12^. The former is ideal for sequencing large genomes and provides high-quality long reads that allow genome reconstruction with an accuracy of more than 99.99%^13^. High-quality assemblies based on PacBio sequences were recently published for *Arabidopsis thaliana* (135 Mb)^13^, *Oropetium thomaeum* (245 Mb)^14^, *Utricularia gibba* (82 Mb)^15^, *Chenopodium quinoa* (1500 Mb)^16^, *Zea mays* (2300 Mb)^17^ and *Helianthus annuus* (3000 Mb)^18^. However, to build chromosome-level genome assemblies, spanning long genomic distances and to order the contigs in the right orientation it is necessary to complement the PacBio system with other technologies. Chicago^®^ and Dovetail™ Hi-C are two recently developed methodologies based on proximity DNA and chromatin ligation that complement the PacBio system and result in an extremely precise sequence assembly, orientating the contigs and increasing the N50 to values that can be as large as 30 Mb^19^. A good example of this integration of technologies is shown by the genome assembly of the tropical fruit, the durian, *Durio zibethinus*, where the N50 was 22.7 Mb^20^.

The availability of a high-quality diploid genome assembly of *E*. *curvula* will contribute to establishing its evolutionary relationships with other members of the Poaceae family, unraveling the taxonomy of the *E*. *curvula* complex, looking for new strategies to improve forage quality and directing the assembly of more complex and heterozygous tetraploid genomes harboring the apomixis control region(s). The elucidation of this region(s) and the associated apomixis genes could lead to revolutionary developments in terms of crop improvement.

To obtain a high-quality genome assembly of the diploid *E*. *curvula* genotype a combination of PacBio long read sequencing with Chicago and Hi-C technology was employed. The final genome assembly had 603 Mb distributed in 1,143 scaffolds, an N50 of 43.4 Mb with 28% of the bases corresponding to repetitive elements and was validated using DArT-seq and Simple sequence repeats (SSR) markers.

## Results

### Sequencing and Assembly

The PacBio Sequel platform was employed to sequence two *E*. *curvula* libraries of 10 and 20 kb resulting in 6,223,627 and 3,309,811 raw reads respectively. The average read length size was 5,296 for the 10 kb and 7,018 for the 20 kb libraries, covering 90.32X of the estimated genome size. The best assembly was obtained using the FALCON software with the following settings: i) a length cut-off of 7,500 bp, ii) overlap filtering parameters of minimum and maximum coverage of 5 and 120X, respectively and iii) a maximum difference of coverage of 120X between the 5′ and 3′ ends. The *de novo* assembly consisted of 3,118 contigs with an N50 of 378,697 bp, representing 97% of the genome length (Table 1). This assembly was later polished using the software Arrow, improving the accuracy per base content and increasing the percentage of complete BUSCO genes from 88.4% to 96% (Table 1). In this assembly 98.88% of the bases were found with a quality score more than 30, this means that 98.88% of the polished genome has an error rate less than 0.001.

**Table 1:** *E*. *curvula* genome assembly metrics.

	PacBio	Polished	Only Chicago	Chicago + Hi-C
Size bp	601,616,585	600,872,314	602,350,000	602,432,814
N-50 bp	378,697	380,299	791,258	43,411,000
#sequences	3,516	3,118	1,884	1,143
Average bp	171,108.24	192,710.81	319,718.81	527,062.82
BUSCO	C:88.4%	C:96.0%	C:96.1%	C:96.4%

The next step was to sequence the Chicago library, achieving 432 million 2 × 150 paired-end raw reads. The combination of these reads with the primary contigs of the FALCON assembly using the Hi-Rise assembler increased the N50 from 0.378 Mb to 0.791 Mb, decreasing the number of sequences to 1,884 (Table 1). Although this strategy vastly improved the contiguity of the assembly, another improvement involved the preparation of a Hi-C library, in which 333 million 2 × 150 bp paired-end raw reads were achieved. Using the Hi-C library as input within the Chicago assembly the N50 increased to 43.41 Mb and the number of scaffolds decreased to 1,143. The final BUSCO results were 96.4%, 80.3% of them were single copy while 16.1% were duplicated.

The pipeline used to sequence and assembly the genome, and the experimental steps in its analysis, are shown in Supplementary Fig. S1.

**Figure 1:**
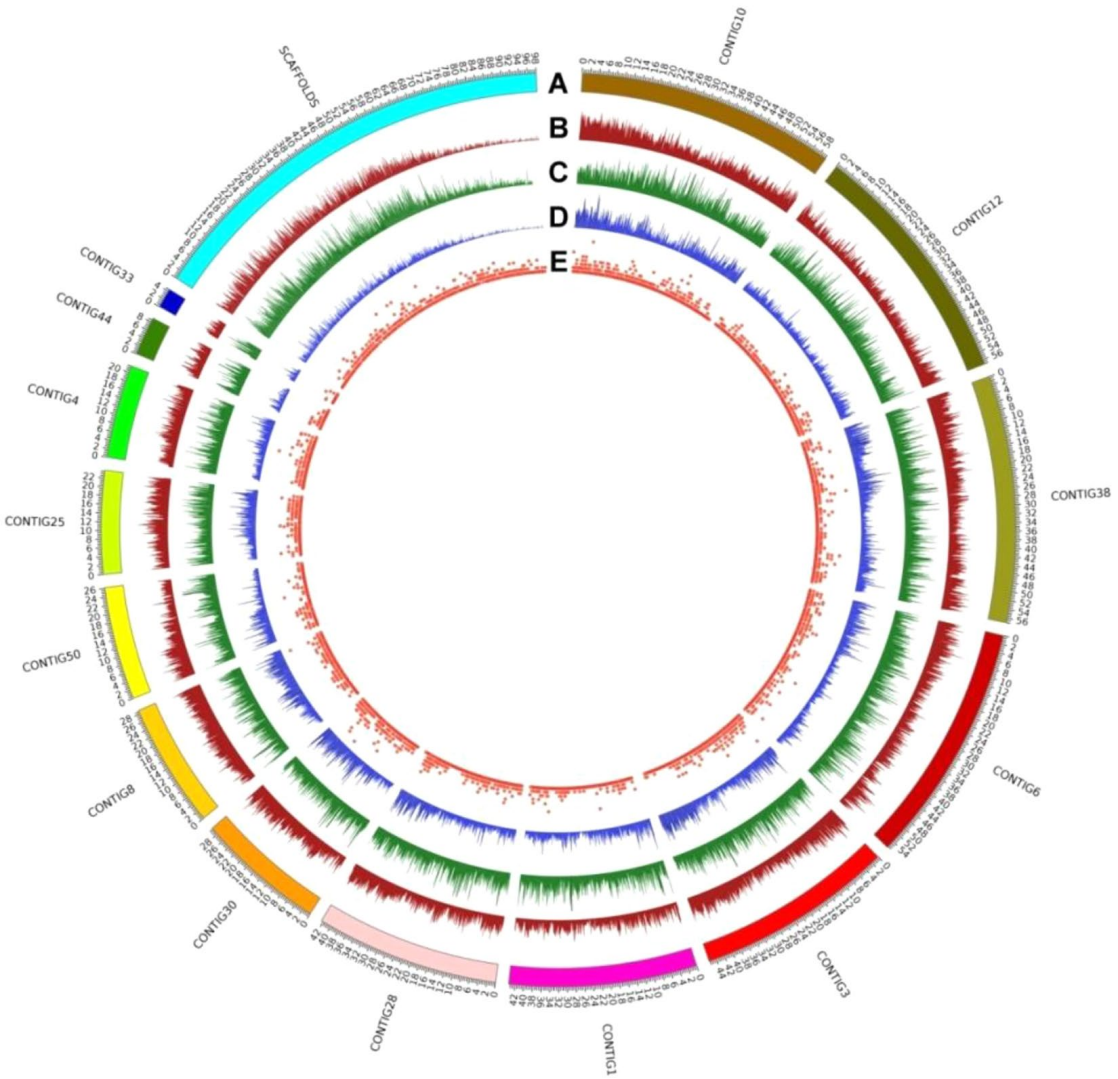
Circos plot of the *E*. *curvula* genome assembly. (**A**) The fourteen longest scaffolds plus one scaffold representing the shortest scaffolds were merged. (**B**) Gene density, (**C**) Repeat elements content, (**D**) DArT reads density and (**E**) DArT marker density.

The *E*. *curvula* genome assembly presented here is one of the few published genomes (NCBI Bioproject PRJNA508722) with a scaffold N50 value greater than 10 Mb, having seven scaffolds with almost the size of complete chromosomes. A high level of contiguity was obtained, since 83.7% of the genome size was contained in the first 14 scaffolds (Fig. 1).

### DArT-seq (Diversity Arrays Technology sequenciation) Analyses and Marker Mapping

**Figure 2:**
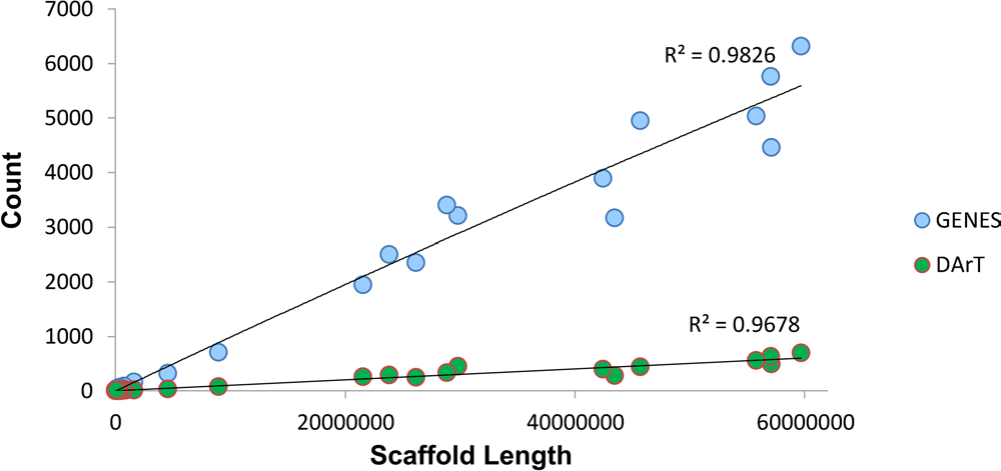
Regression between number of gene models (light blue circles) and number of DArT markers (green circles) and the *E*. *curvula* scaffold length. Each circle represents a scaffold. The regression analysis shows that gene and DArT marker counts  are directly proportional to  the scaffold size, meaning that the largest scaffold, the higher the gene and DArT count.

DArT is a recently developed technology for SNP markers discovery based on the reduction of genome complexity. A total of 6,027 (95.5%) of the original 6,307 SNPs markers sequences present in cv. Victoria were successfully aligned to the genome assembly. The longest scaffolds present a proportionally higher number of markers than the shortest ones and the same tendency was appreciated in the gene number (Supplementary Table S1, Fig. 2).

DArT reads density in the chromosome-scale scaffolds was used to validate the assembly. DArT libraries are designed to avoid repetitive elements and target active regions covered predominantly by genes. A higher number of genes is expected in the distal arms regions than in the central regions. In fact, the seven longest scaffolds showed higher read density at the 3′ and 5′ ends than in the central region (Supplementary Fig. S2). Thus, contigs with the longest length, the greatest number of genes and DArT markers could be complete chromosomes.

### Repetitive Elements and Gene Annotation

**Figure 3:**
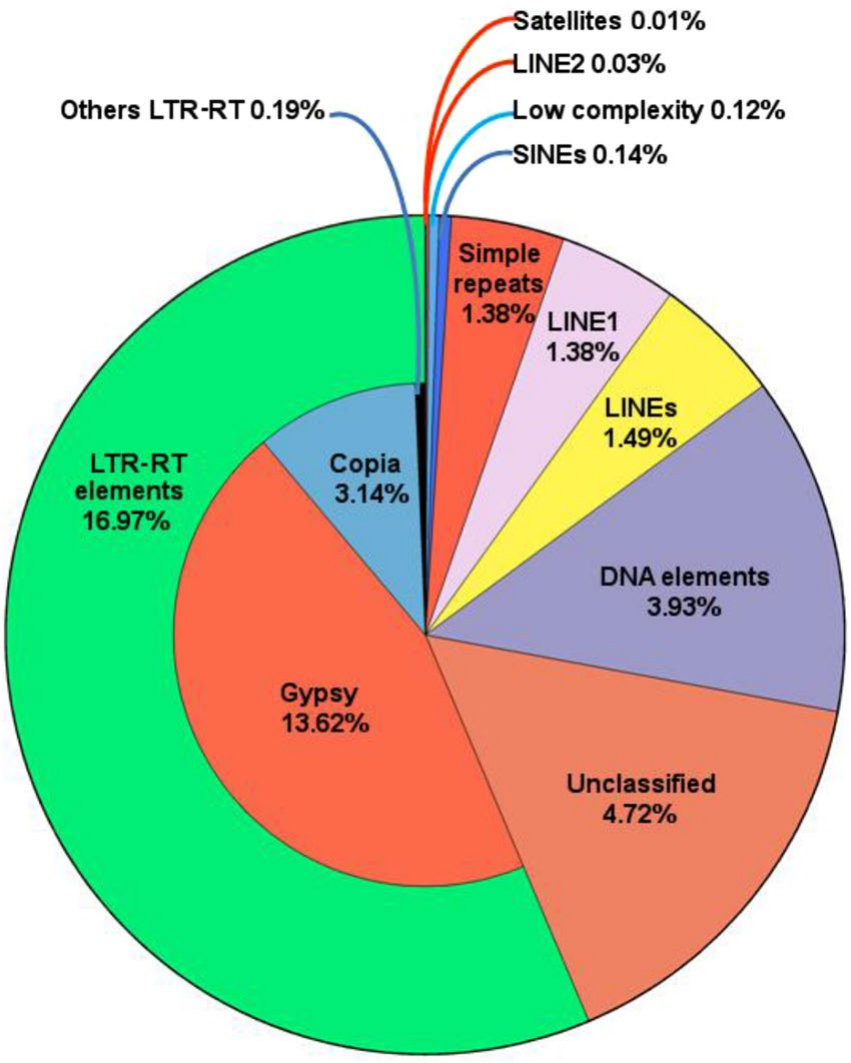
Percentages of repetitive elements present in the *Eragrostis curvula* genome assembly. Each color represents a different class of element. The total content of repetitive elements present in the assembly was 28.7%.

The analyses over the *E*. *curvula* genome assembly established that 28.7% is composed by repetitive elements, mainly Long Terminal Repeats retroelements (LTR-RT) (16.97%), followed by DNA and unclassified elements (Fig. 3 and Supplementary Table S2), as seen in most of the grasses^21^. Within the most representative LTR-RTs were the *Gypsy* and *Copia* superfamilies, accounting for 13.62% and 3.14% of the total, respectively, with the ratio between them of 4.3:1. This value is very close to the one found in *E*. *tef* (4.27:1)^22^ and higher than the corresponding one in *S*. *italica* (3.08:1)^23^, *Z*. *mays* (1.91:1)^17^ and *S*. *bicolor* (3.67:1)^24^ (Supplementary Table S3). Previously reported ESTs related to repetitive elements present in *E*. *curvula*´s floral and leaf libraries^25^ were mapped onto the genome assembly. Using this scheme a LTR structure of a *Gypsy* superfamily of retrotransposons identified from the EST EH191456 was validated, since it was present in at least 16 scaffolds (Supplementary Table S4).

**Figure 4:**
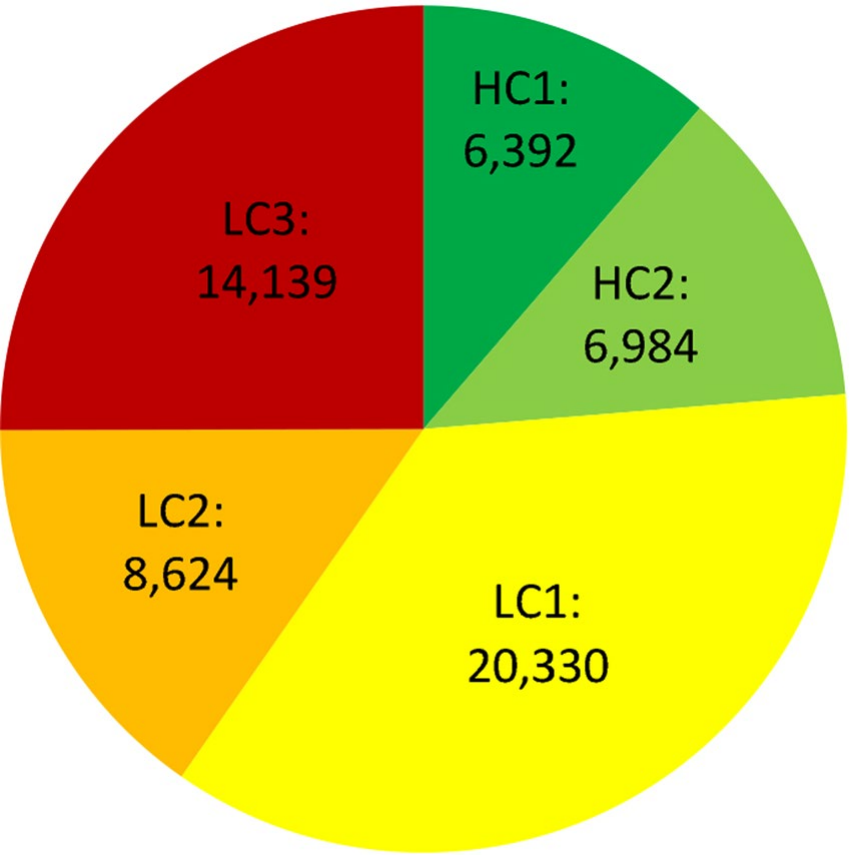
Number of High Confidence (HC) and Low Confidence (LC) gene models present in the *E*. *curvula* genome assembly. The total number of predicted genes was 56,469. LC1 plus HC is approximately the number of genes expected for the genome.

Gene annotation was performed using an *ab initio* prediction algorithm combined with data from ESTs and RNA-seq databases from different tissues of *E*. *curvula* and from proteins of related species. After three iterations of the MAKER software, 56,469 gene models were obtained with an average size of 1,424 bp and 93.4% of the complete BUSCO genes (Supplementary Fig. S3 and Supplementary Table S5). These genes were classified into two main categories: High Confidence (HC) and Low Confidence (LC) genes, then divided into two and three subcategories, respectively. Using this strategy 13,376 HC genes and 20,330 LC1 genes were identified, representing approximately the number of genes expected for the species (Fig. 4). The protein domains were inferred using the InterProScan software (Supplementary Table S6), finding 35,713 matches in the Pfam domain database. Gene ontology analysis based on 56,469 genes classified 33,601 genes into biological processes, 17,710 into cellular components and 33,820 into molecular function, finding 29,462 genes with at least one GO annotation category (Supplementary Fig. S4).

### *Eragrostis curvula* Genome Evolution Among the Poaceae Family

**Figure 5:**
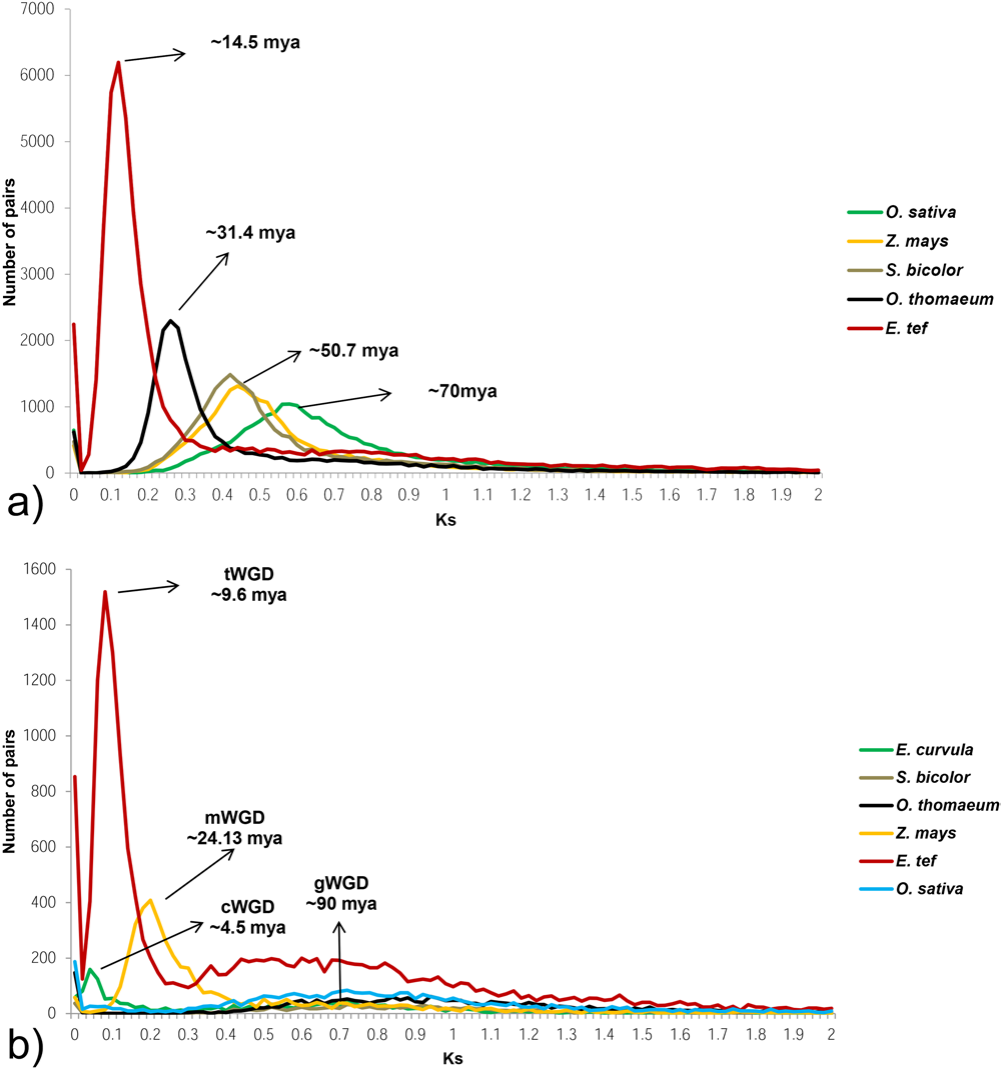
(**a**) Distribution of the estimated synonymous substitutions rate (Ks) between *E*. *curvula* and the selected Poaceae orthologous genes. Peaks represent the divergence between *E*. *curvula* and the selected species. (**b**) Distribution of the synonymous substitution rate within each selected genome paralogous genes. The whole genome duplication shared by all the grasses (gWGD) was estimated to occur 90 Mya. The peaks represent the tetraploidization events of *E*. *tef* (tWGD) *E*. *curvula* (cWGD) and *Z*. *mays* (mWGD).

**Figure 6:**
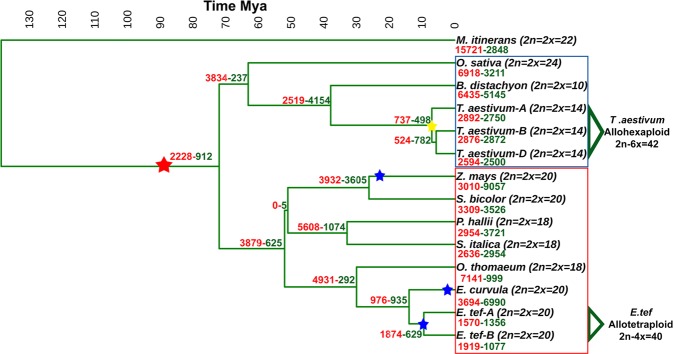
Maximum likelihood phylogenetic tree of selected Poaceae species based on 1,185 orthogroups. The time scale above the tree indicates the evolution time in millions of years ago (Mya). The numbers in red in the nodes show the number of contracted gene families while the numbers in green refer to the number of expansions. The ancient WGD (red star) was calculated to happen around ~90 Mya. The *Z*. *mays*, *E*. *tef and E*. *curvula* tetraploidization events (blue stars) were placed at 24.1, 9.6 and 4.5 Mya, respectively. The yellow star represents the hexaploidization of *T*. *aestivum*. C4 grasses species are included in the red box and C3 in blue box.

The duplication events of the Victoria cv. genome over the time has been evaluated through a paralogous genes analysis (Supplementary File 1). The Ks (synonymous substitutions rate) peaks show two paleopoliploidy events across the time, the ancestral paleoduplication event shared by all the grasses estimated that have occurred 80–90 Mya (Millions the years ago) and a recent (4–5 Mya) duplication before the *in vitro* culture diploidization of Victoria cv. (Fig. 5). Since the ancestral whole genome duplication a high contraction rate over the time was observed. In the BEP (Bambusoideae, Ehrhartoideae, and Pooideae) divergence from the PACCAD (Panicoideae, Arundinoideae, Chloridoideae, Centothecoideae, Aristidoideae, Danthonioideae) a 2.44 contraction/expansion rate was observed (Fig. 6). After that, the corresponding rate in the Panicoideae-Chloridoideae divergence was 6.2. Then, in the divergence between *Eragrostis* and *Oropetium* the rate increased up to 16.88. The contraction rate for *O*. *thomaeum* genome assembly was 7.14 being the expansion higher than the contraction in all the evolution history after the grasses whole genome duplication (gWGD). More recently, during the *Eragrostis* speciation the rate was stabilized in 1.04 and after the *E*. *tef* allotetraploidization (9–10 Mya) the rate increased again to 2.97. Correspondingly, *E*. *curvula* present an allotetraploidization event 4–5 Mya. However, it is not possible to calculate the gene loss after tetraplodization because one copy of the genome was lost during the *in vitro* diploidization event. The high proportion of expansions in *E*. *curvula* could be related to the low confidence genes models.

The gene models from selected monocots species, such as *E*. *tef* (A and B genome), *Triticum aestivum* (A, B and D genome), *Oryza sativa*, *Z*. *mays*, *S*. *bicolor*, *S*. *italica*, *Panicum hallii*, *O*. *thomaeum*, *B*. *distachyon* and *Musa itinerans* were grouped in orthogroups with the *E*. *curvula* genes models. The total number of defined orthogroups was 24,747, with 9,189 groups shared by the eleven species (Supplementary Fig. S5). To assess the evolutionary relationships within the subfamily we grouped them into Chloridoideae, Panicoideae, Pooideae and Ehrhartoideae. The number of gene families shared by the Chloridoideae subfamily was 12,940, while the number of genes families that are in common within the Panicoideae and Pooideae subfamilies were 13,621 and 15,532, respectively (Supplementary Fig. S6). Among the C4 grasses 11,570 common orthogroups were identified between both subfamilies, whereas 2,051 and 1,370 were found to be specific to the Panicoideae and the Chloridoideae subfamily, respectively. When an analogous analysis was performed with the C3 species, 14,203 shared orthogroups were identified, whereas 1,329 and 2,601 orthogroups were unique to the Pooideae and the Ehrhartoideae subfamily, respectively.

When the assembled genomes sharing orthogroups were analyzed, we found that the second most abundant group of species sharing orthogroups was constituted by a combination of two species (3,991 orthogroups). The combination of *E*. *tef* and *E*. *curvula* contributed to this group with 616 orthogroups, representing 15.5% of the total (Supplementary Fig. S5). This means that 616 orthogroups were shared by these two species exclusively being *E*. *tef* the most closely related species to *E*. *curvula*. This was confirmed by the construction of a phylogenetic tree with the eleven species, in which the divergence between *E*. *curvula* and *E*. *tef* was calculated as occurring 14.5 Mya and, as was expected, *E*. *curvula* was located close to the other C4 grasses (Fig. 6).

**Figure 7:**
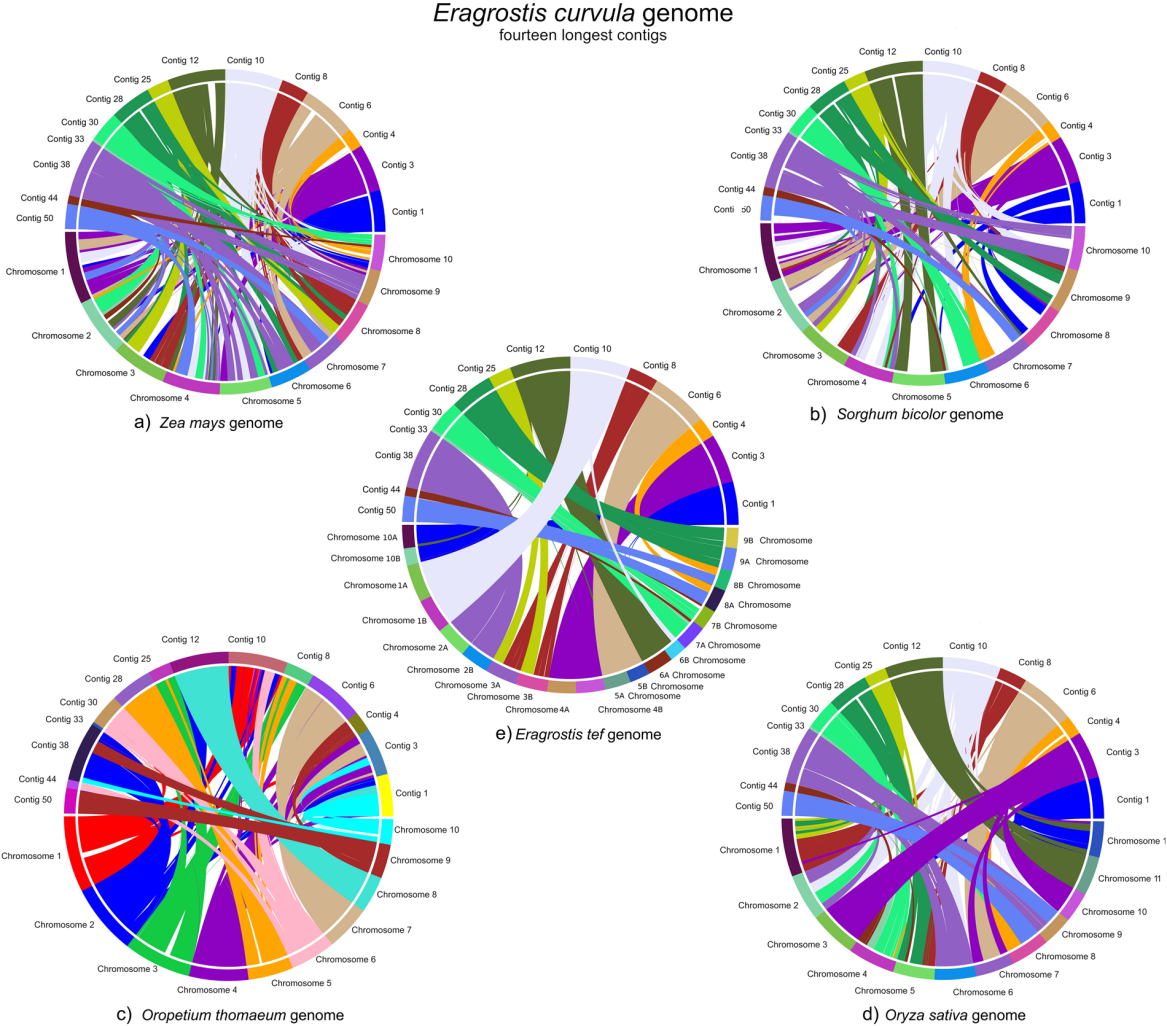
Synteny between *E*. *curvula* and: (**a**) *Zea mays;* (**b**) *S*. *bicolor;* (**c**) *O*. *thomaeum* and (**d**) *O*. *sativa* genome assemblies. In *E*. *curvula* the fourteen longest scaffolds are represented. Seventy nine percent of the *E*. *curvula* genome was covered by the *Z*. *mays* and *S*. *bicolor*, 85% by *O*. *thomaeum* and 84% by *O*. *sativa* genomes, respectively.

**Figure 8:**
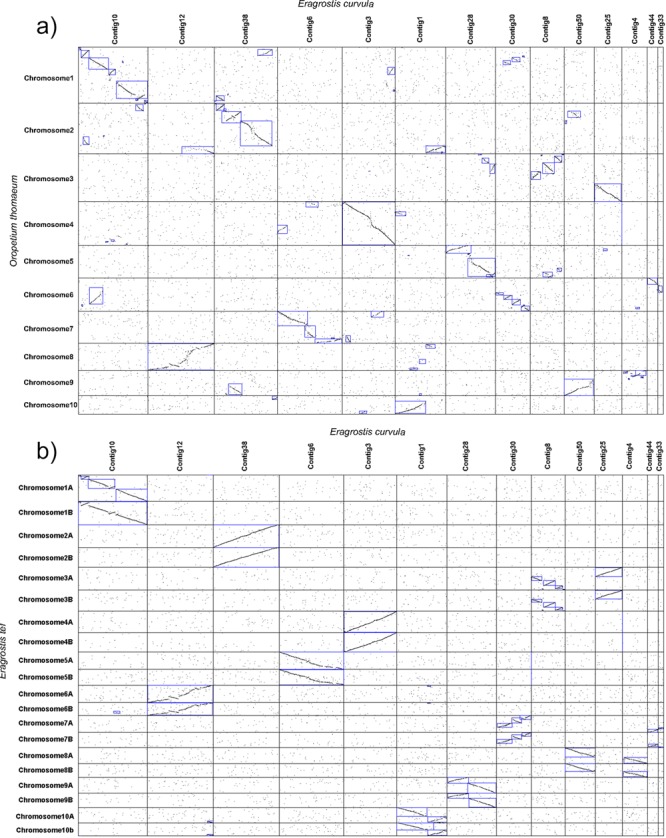
Syntenic dotplot between *E*. *curvula* scaffolds and *O*. *thomaeum* (**a**) and *E*. *curvula* scaffolds and *E*. *tef* (**b**) genomes. The black dots indicate syntenic genes between the species. Blue shapes represent syntenic blocks between the species. Contigs 10, 12, 38, 6 and 28 constitute  complete chromosomes in both species but the number of chromosome rearrangements is lower between *E*. *curvula* and *E*. *tef*.

Using SyMAP to plot the syntenic regions between *E*. *curvula* and the other monocots species it was found that 79% of the *E*. *curvula* genome assembly length is covered by the *Z*. *mays* and *S*. *bicolor* syntenic blocks (Fig. 7, Supplementary Table S7). These analyses also revealed the presence of 182 reverse blocks between *E*. *curvula* and *Z*. *mays* and 149 between *E*. *curvula* and *S*. *bicolor*, representing genome rearrangements that occurred during the evolution of these grasses. Despite the divergence, 98% of the *O*. *sativa* assembly was covered by *E*. *curvula* scaffolds, sharing 262 syntenic blocks. Interestingly, *O*. *sativa* chromosome 3 is fully covered in the same orientation by the *E*. *curvula* Contig 3 (Fig. 7), indicating the close conservation of this chromosome among these grasses. The syntenic analysis over the *O*. *thomaeum* genome assembly revealed that 96% of the genome was covered by the *E*. *curvula* scaffolds (Supplementary Table S7). However, different patterns were observed between the *E*. *curvula* scaffolds and the *O*. *thomaeum* chromosomes (Fig. 8). For example, *E*. *curvula* Contig 3, that completely covers *O*. *sativa* chromosome 3, also covers the entire *O*. *thomaeum* chromosome 4, whereas *O*. *thomaeum* chromosome 3 is completely covered by *E*. *curvula* Contigs 25 and 8, suggesting that these two *E*. *curvula* scaffolds constitute one single chromosome. Other *O*. *thomaeum* chromosomes, such as 1, 2, 4, 5, 7, 8 and 10, are fully covered by Contigs 10, 38, 28, 6, 12 and 1, respectively, even when several rearrangements were detected.

The latest *E*. *tef* genome assembly version^26^ ratifies the chromosome scale of the Contigs 10, 12, 38, 6, 3, 1, 28 and provides evidences that the pairs of Contigs 8–25, 50–4, and 30–44 correspond to the *E*. *tef* chromosomes 3, 8 and 7 respectively, thus, assuming that there are not changes in the chromosome structure the ten *E*. *curvula* chromosomes seem to be present in the assembly with a high level of contiguity.

### Genetic Relationships Among *E*. *curvula* Genotypes Assessed by SSR Analyses

SSR specific primers previously designed from transcriptomic sequences^9^ were mapped onto the *E*. *curvula* genome assembly. Twenty eight out of the 35 reported *E*. *curvula* SSRs primers (Supplementary Table S8) gave 100% identity and 100% of coverage with the *E*. *curvula* genome assembly. Additional SSR primers were designed based on the cv. Victoria assembly. Regarding the newly designed primers over the Victoria genome 14 out of 15 amplified with the expected amplicons size (Supplementary Fig. S7).

**Figure 9:**
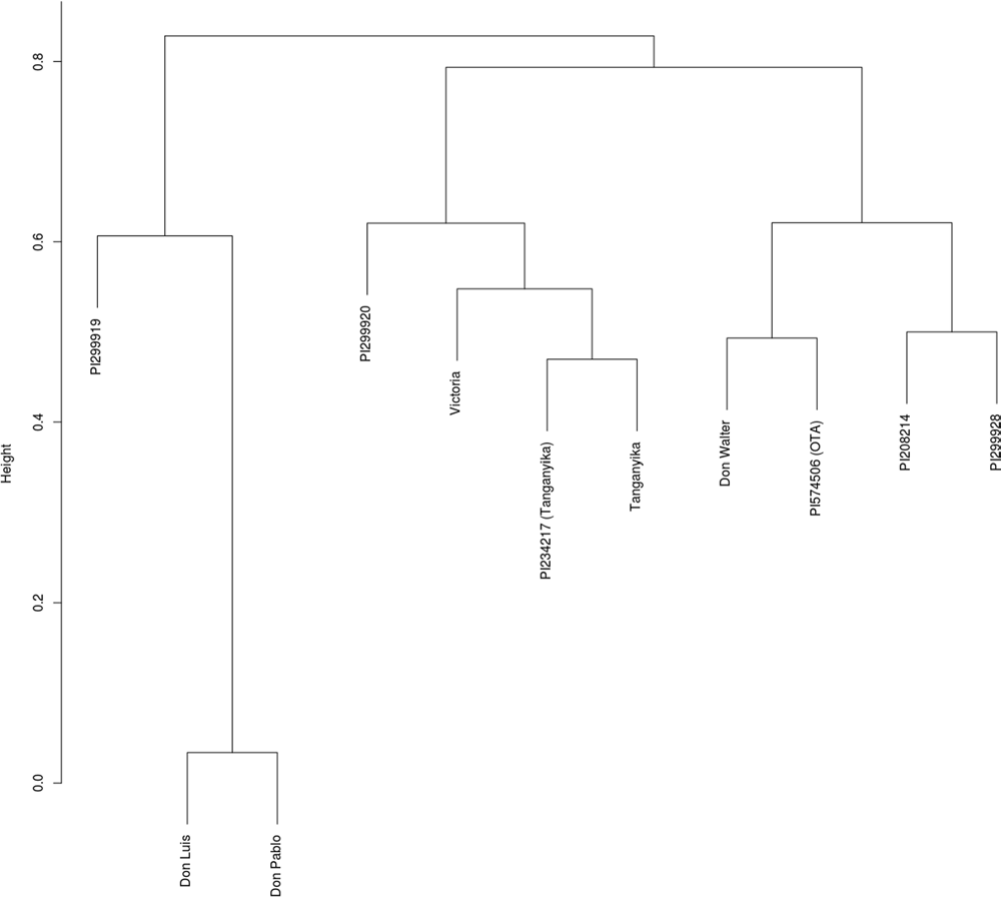
Phylogenetic tree of different cultivars of *E*. *curvula* constructed using the Jaccard distance matrix calculated from SSR markers. Cultivar Victoria was placed next to the diploid cultivar PI299920 and the tetraploid Tanganyika INTA. The diploidization of this cultivar through *in vitro* inflorescence culture gave rise to the Victoria cv.

A phylogenetic tree was constructed using the Jaccard distance matrix calculated from the SSR markers (Fig. 9). This tree grouped the heptaploid cultivars Don Luis and Don Pablo together, showing the similarity between each other and the divergence from the other cultivars. Cultivar Victoria was located close to the diploid PI299920 and in the same branch as the tetraploid cultivars Tanganyika INTA and Tanganyika USDA. Tanganyika INTA is an apomictic tetraploid cultivar that gave rise to cv. Victoria through chromosome reduction by *in vitro* inflorescence culture^27^. Due to its similarity with cv. Victoria, it is a candidate for sequencing to identify the genomic region controlling apomixis.

### Lignin Pathway

Several reports have established that digestibility in forage grasses can be improved through downregulation of genes involved in the lignin pathway by genetic engineering^28–30^. Therefore, sequence information of lignin biosynthetic genes and their controlling elements is crucial to manipulate their expression. Using the KEGG^31^ database 16 gene models (Supplementary Fig. S8) were found to be involved in the *E*. *curvula* lignin biosynthesis pathway, from the first gene, corresponding to phenylalanine ammonia-lyase, to the final products, guaiacyl (G), p-hydroxyphenyl (H) and syringyl lignin (S) monolignols.

From this pathway we focused on the enzyme caffeoyl shikimate esterase (CSE) because of its recently identified role in lignin biosynthesis^32,33^. This enzyme affects the production of caffeoyl-coenzyme A 3-O-methyltransferase, previously studied by our group in *E*. *curvula*^34^ which could be used together with CSE to improve forage quality. Genes encoding class II enzymes are widespread in the plant kingdom, while genes encoding class I CSE enzymes are not present in all species. Aligning class I CSE from *O*. *sativa* and class II from *S*. *bicolor* against the *E*. *curvula* genome assembly we could identify both gene CSE classes. CSE genes were amplified from *E*. *curvula* using specifically designed PCR primers (Supplementary Table S9), and the resulting amplicons were cloned and sequenced, obtaining a perfect match with the sequences from the genome assembly. Moreover, our previous transcriptomic analysis^9^ showed the presence of mRNA from both genes, indicating that they are actively transcribed in this species.

The sequences of *E*. *curvula* class I and II genes were aligned to the corresponding sequences from other members of the Poaceae family (Supplementary Table S10). The two classes were found in *O*. *thomaeum*, *P*. *hallii* and *O*. *sativa* and only class II was present in *Z*. *mays*, *S*. *bicolor*, *S*. *italic*, *B*. *distachyon* and *T*. *aestivum*. We found single equal-sized amplicons in *E*. *tef* and *E*. *curvula* for class I CSE (Supplementary Fig. S9). Cloning these amplicons confirmed the existence of CSE class I in both species (Supplementary Fig. S10). Looking into the evolution of the grasses (Fig. 6) it is possible to deduce that the loss of the CSE class I occurred at different times during the divergence of these species, since the enzyme is absent from the Pooideae subfamily, present in the Chloridoideae and appears only in some members of the Panicoideae.

### WRKY Transcription Factor Family

The WRKY transcription factor family is one of the most studied gene families associated with biotic and abiotic stresses in plants. Since *E*. *curvula* is a species adapted to high temperature, high radiation and drought stress, classification of its WRKY transcription factors is central to understand the mechanism(s) involved in its tolerance to these conditions.

**Figure 10:**
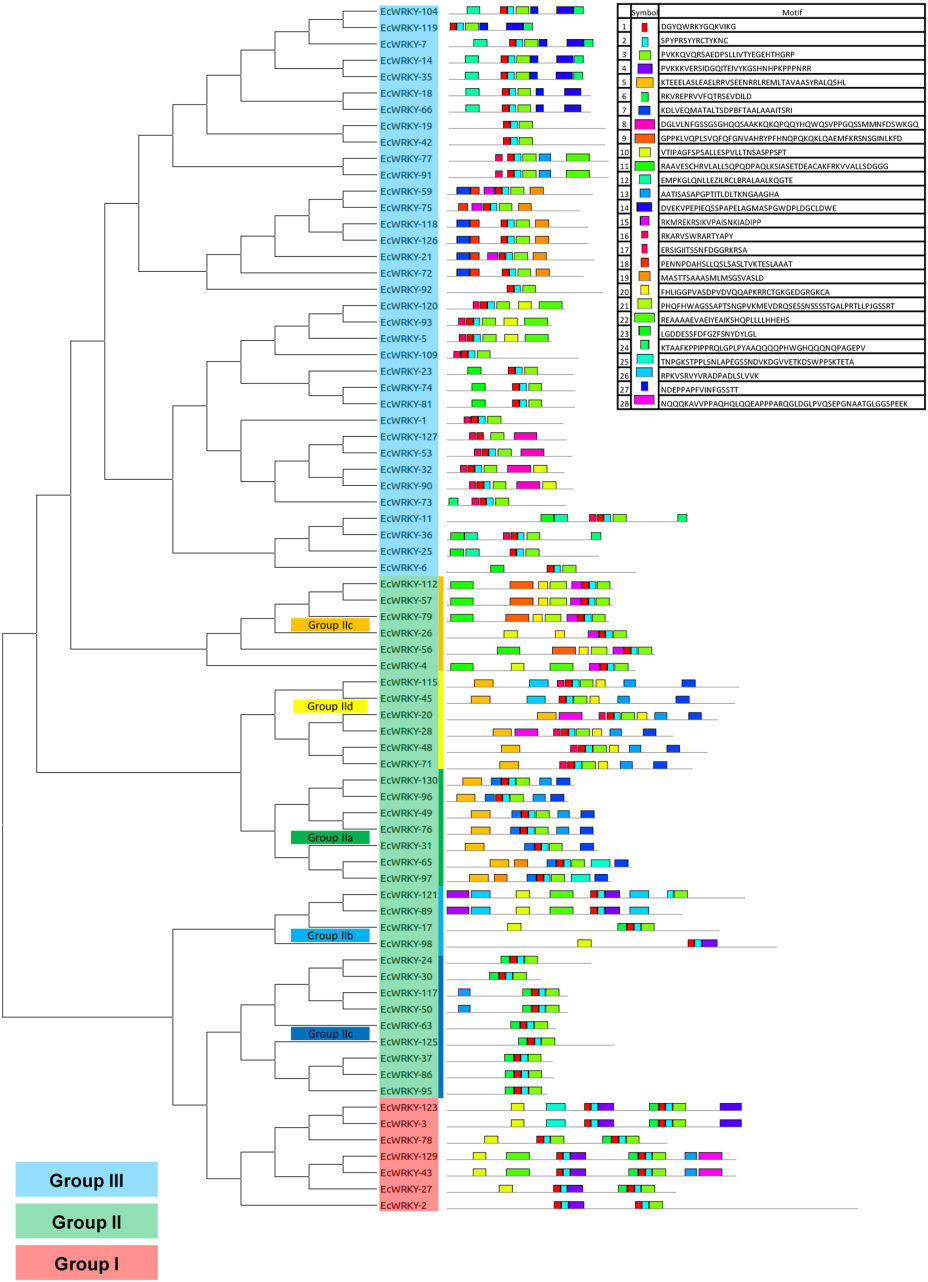
Phylogenetic tree and sequence motifs of the *E*. *curvula* WRKY transcription factor family. Bars with the same shape and color represent the same motif. Seven proteins were classified as group I (red), 32 as group II (green) and 35 as group III (blue).

From the Pfam annotated gene models, 74 genes with WRKY and zinc finger motifs were found (Supplementary Fig. S11). Seven out of the 74 were classified as group I, 32 as group II and 35 as group III. Group II was divided into five subclasses according to the remaining sequence motifs and the phylogenetic distribution (Fig. 10). The EcWRKY family has 39 of its members clustered into 13 genomic regions of less than 100 kb while other members are isolated (Supplementary Table S11). This spatial distribution agrees with previously reported data for other grass species^35–37^.

## Discussion

Here we present the first high-quality genome assembly of a diploid genotype of *E*. *curvula*. This diploid assembly is a starting point for the genome assembly of the most complex polyploids of the same genus, which harbor the region(s) that controls diplosporous apomixis, and may allow us to assess the complex relationship between apomixis and ploidy.

The final FALCON assembly, after polishing, rendered an N50 of 0.380 Mb, 3,118 contigs and 96% of complete BUSCO genes. At this point, due to the size, the complexity and repetitiveness of the *E. curvula* genome, we could not achieve the assembly metrics reached by other plant genomes based on PacBio assembly alone. However, the N50 of our assembly was higher than the ones obtained for other grasses using other sequencing technologies, as was the case for *Aegilops tauschii* (4.3 Gb)^38^, *Triticum urartu* (4.94 Gb)^39^ and *S*. *Italica* (490 Mb)^23^ with N50 values of 0.207 Mb, 0.064 Mb and 0.254 Mb, respectively.

The promising results obtained with the FALCON assembly encouraged us to look for a scaffolding technology to increase genome assembly contiguity. One of the latest methodologies to obtain chromosome-scale scaffolds is the proximity ligation-based technology used by Chicago and Hi-C. The combination of these technologies with the FALCON assembly increased the N50 to 43.41 Mb and decreased the number of scaffolds to 1,143. The only currently available report about this combination of technologies is for *D*. *zibethinus* (738 Mb)^20^, in which an N50 of 22.7 Mb was achieved, half of the final *E*. *curvula* N50. Other assemblies using a different combination of technologies such as optical mapping and Hi-C, for *Chenopodium quinoa* (1.45 Gb)^16^, *M*. *truncatula* (465 Mb)^40^ and *Arabis alpine* (370 Mb)^41^, rendered N50 values of 3.84 Mb, 12.5 Mb and 31 Mb, respectively. A combination of optical mapping and Chicago led to an N50 of 95 kb in *Hevea brasiliensis* (2.15 Gb)^42^. In *Manihot esculenta* (1.23 Gb)^43^ Chicago only was used with a resulting N50 value of 27.7 kb. These results show that the combination of Hi-C and Chicago is very powerful, increasing the N50 more than other combinations of technologies.

Our final *E. curvula* genome assembly has 96.4% of complete BUSCO genes, covers 97% of the estimated genome length and contains 95.5% of the DArT markers, all this components suggest the near completeness of this assembly.

An important feature of grass genomes is the presence of repetitive elements that differ in number and complexity among species, being higher in more complex genomes^44,45^. In the *E*. *curvula* genome assembly we found 28.8% of repetitive elements, a similar value to the 27.46% reported for *E*. *tef*^22^ and 21.4% for *B*. *distachyon*^46^, but lower than the 46.44% found in *S*. *italica*^23^ and the 62% from *S*. *bicolor*^24^.

The number of gene models found in *E*. *curvula* was 56,469. The BUSCO analysis performed on these models resulted in 28.1% of duplicated BUSCO genes. This overestimation seems to occur in most species, for example, the recently published *T*. *aestivum* genome assembly^47^ has 259,979 gene models, a very high number of genes even for an hexaploid species. From the gene models, we assessed the evolution of *E*. *curvula* within the Poaceae family, positioning the species close to *E*. *tef* and finding 9,189 orthogroups shared by the selected Poaceae subfamilies.

Seventy-four WRKY transcription factors were identified in the *E*. *curvula* genome assembly, and classified into three main groups and five subgroups, based on the results obtained by other researchers^48^. The identification, classification and characterization of this gene family constitute a key step into the elucidation of the molecular basis of the drought tolerance of this species^49^ since it allows the design of further specific expression studies that would contribute to dissect their involvement in this important trait of *E*. *curvula*.

One of the main limiting factors of *E*. *curvula* as a forage source it is its low digestibility^50^ a trait that has the potential to be improved by transgenic modification^30^. Two classes of CSE, an enzyme involved in lignin reduction, have been described^32,33^, with class II present in all species examined, whereas class I is only present in some of them. Interestingly, class II was present in all the evaluated Chloridoideae species, such as *O*. *thomaeum*, *E*. *tef* and *E*. *curvula*, finding not previously reported (Supplementary Table S10).

In conclusion, using a combination of different technologies to assemble and validate the *E*. *curvula* genome a notable advance in the *de novo* assembly of non-model genomes was achieved. This assembled genome also provides an invaluable tool to find new targets for crop improvement regarding classical focused traits, such as drought tolerance and digestibility. Finally, the availability of this assembly provides the foundation for the assembly of more complex tetraploid apomictic *E*. *curvula* genomes, aiding in the study of the reproductive mode.

## Methods

### Plant Material

DNA for genome sequencing was extracted from *E*. *curvula* cv. Victoria, a sexual diploid (2n = 2x = 20) genotype obtained from *in vitro* culture of inflorescences of cv. Tanganyika^27^ and registered at the National Cultivar Register, Argentina (UNST1122, RC9192). This accession couldn’t be selfed to decrease the heterozygosity because its self-incompatibility. Leaf samples for DNA extraction were collected from a plant growing in the greenhouse at CERZOS, CCT – CONICET Bahía Blanca, Argentina. DNA for DArT markers was extracted from leaves of a mapping population consisting of 63 individuals derived from the cross between two tetraploid *E*. *curvula* cultivars, OTA x Don Walter, and from two samples of cv. Victoria. DNA for SSRs amplification was extracted from leaves of different *E*. *curvula* cultivars: USDA accessions PI208214 (2x), PI299919 (2x), PI299920 (2x), PI299928 (2x), PI574506 (OTA, 4x), PI234217 (Tanganyika, 4x), and cultivars from INTA germplasm collection Tanganyika (4x), Don Walter (4x), Don Pablo (7x) and Don Luis (7x) (Supplementary Table S12).

### DNA Extraction

DNA samples for PacBio long reads and DArT sequencing were obtained from 80 mg of fresh leaf tissue using a CTAB-based method^51^. The protocol was adapted to obtain large DNA molecules (gDNA), taking care in the critical steps to avoid breaking, resulting in an average length of 41,800 bp and a concentration of 0.7880 ng/μL.

For the Chicago and Dovetail Hi-C proximity ligation libraries, fresh leaf tissue from the same plant was delivered to Dovetail Genomics (www.dovetailgenomics.com) for extraction and sequencing of pure DNA and endogenous chromatin using a proximity ligation-optimized Dovetail in-house protocol.

### Library Preparation and Sequencing

Long-read sequencing of cv. Victoria DNA was performed using Pacific Bioscience’s Single Molecule Real-Time (SMRT) chemistry through the Sequel platform (www.pacb.com) at the University of Liverpool Centre for Genomic Research (UK). Current PacBio systems generate reads with an average size of nearly 20 kb and a maximum length of over 60 kb. Two Libraries of 10 kb and 20 kb were  prepared through the BluePippin (Sage Science) fragment selection method (http://www.sagescience.com/products/bluepippin/). After repairing the ends and an adapter ligation process the libraries were sequenced. The coverage of the estimated haplotype (620 Mb) was 90X, 10X greater than the one recommended for this technology for a genome with characteristics such as that of *E*. *curvula* (www.pacb.com/calculator-whole-genome-sequencing/).

To improve the assembly contiguity and to orientate the contigs, Chicago and Dovetail Hi-C libraries were sequenced starting from endogenous chromatin and high molecular weight DNA, respectively. The Chicago and Dovetail Hi-C libraries were sequenced through a 2 × 150 paired-end Illumina HiSeq2500 platform.

### Genome Assembly

The PacBio raw reads were assembled with FALCON^52^ and Canu^53^ software exploring different parameters (Supplementary Table S13). The assembly quality was assessed by comparing numerous metrics (N50, assembly size, number of contigs and average contig length). The assembly was also evaluated using BUSCO v.3^54^. This software uses a large selection of widespread orthologous single-copy genes as benchmarks to gauge the completeness of the novel assembled genome. The assembly with the highest N50, the least number of contigs, the highest average contig length and with more complete, less fragmented and/or missing BUSCO genes, was chosen.

After this procedure, the PacBio SMRT tool reference guide (https://www.pacb.com/wp-content/uploads/SMRT-Tools-Reference-Guide-v4.0.0.pdf) was followed to polish the draft genome assembly. The raw reads were aligned with the pbalign software against the assembly with the following criteria: minimum alignment length 50 bp; minimum similarity and minimum accuracy, 70%. The pbalign output was used as input for the Arrow software with the default parameters to polish the assembly by choosing the base with the highest coverage in each position.

The final assembly was obtained by scaffolding the polished genome assembly with the data obtained through the Chicago and Dovetail Hi-C libraries. The Dovetail Hi-Rise scaffolding software^55^ was used to integrate the data obtained from the Chicago library with the PacBio polished assembly. Finally, the Chicago assembly was combined with the Dovetail Hi-C files through Dovetail Hi-Rise to obtain scaffolds in ranges up to the size of whole chromosomes.

### Diversity Arrays Technology (DArT) Validation

The entire DArT preparation procedure, including SNP calling, was provided by the Genetic Analysis Service for Agriculture Laboratory (SAGA, CIMMyT, México) using an in-house protocol. DArT technology uses the combination of *Pst*I and *Mse*I restriction enzymes in order to reduce the genome complexity. The process separate low copy sequences from the repetitive fraction of the genome (https://www.diversityarrays.com). The fragments obtained from the enzyme digestion were sequenced using the Illumina platform and the reads were used as input by the service provider protocol to *de novo* obtain the markers of 69 bp containing the SNP. The reads and markers are available under the NCBI bioproject PRJNA508722 (https://www.ncbi.nlm.nih.gov/bioproject/) with the SNP position and polymorphism present in the header of each marker sequence. The SNP markers were mapped onto the whole genome assembly using the Bowtie software^56^ with the end-to-end and -k 10 (up to 10 distinct valid alignments for each read) parameter to validate the final assembly with data from another source. Since the DArT libraries are designed to target active regions of the genome the 492,378 DArT reads were mapped with Bowtie onto all the scaffolds using the –k 10 and end-to-end parameter and the hits were plotted with a 500 kb window size.

### Repetitive Sequence Assessment

Repetitive sequences were assessed through three different approaches. The first one was based on the generation of a *de novo* library by the RepeatModeler^57^ software, that uses a modeling package to *de novo* identify repeat families. The second approach uses the TransposonPSI software^58^ that identifies repetitive elements based on the homology to protein or nucleic acid sequences to proteins encoded by diverse families of transposable elements. The result of each program is merged with USERCH v7^59^ taking only a single record when the repetition is included in both programs. The merged file is then classified with the RepeatClassifier script (included in the RepeatModeler package) according to the structure and the type of element present in the sequence in one of the main classes of repetitions (ALU, LINE, LTR, DNA elements and Unknown elements). The sequences were classified to subclass and superfamily level depending if they were complete or not. The final approach consisted of finding homologous repetitive elements in related monocot species. RepBase23, a reference database of repetitive DNA sequences from different eukaryotic species^60^, was used to find homologous sequences. Here, consensus sequences of large families and subfamilies of repeats from *Z*. *mays*, *S*. *bicolor* and *Oryza sativa* were used.

The previously reported ESTs^25^ related to repetitive elements were aligned to the repetitive elements present in the assembled *E. curvula* genome in order to find the complete structure of these elements using the BLASTn algorithm with an e-value of 1.0 e^−10^.

### Gene Annotation

In order to annotate the gene models present in the *E*. *curvula* genome assembly, the repetitive DNA was masked using the RepeatMasker software^61^ using the *de novo* and the homology-based fasta files with the library (−lib) parameter. The -s parameter was used to increase sensitivity in the masking process. To find homology, RMblast, a RepeatMasker-compatible version of the standard NCBI BLASTn program, was used. The main difference between these two programs is that RMblast is optimized to compare the RepeatMasker matrix.

After this procedure two different approaches were used to annotate the genes; the first consisted of the alignment of the genome scaffolds against the *E*. *curvula* floral transcriptome^9^ and ESTs^62^, and protein data from related species like *Eragrostis tef* ^8^, *S*. *italica*^23^, *S*. *bicolor*^24^ and *Z*. *mays*^17^. This analysis was performed using the Exonerate software^63^, a general tool for sequence comparisons, setting the minimum alignment coverage to 80% and the minimum identity to 85%. The second method included *ab initio* gene prediction, an intrinsic method based on gene content and signal detection in which the genomic DNA sequence alone is systematically searched for certain tell-tale signs of protein-coding genes. For this, we used AUGUSTUS^64^ an HMM-based (Hidden Markov Model) gene finder and SNAP software^65^. In *ab initio* prediction is necessary to train the programs in order to create the best model to precisely find the genes. For this purpose, the model used to find the complete genes was extracted from the output of the BUSCO software.

The annotation was performed through the MAKER software^66^, using as input the RepeatMasker output and the protein and RNA alignment obtained from Exonerate in a splice-aware fashion to accurately identify splicing sites. MAKER also uses the gene models predicted by AUGUSTUS and SNAP, compares all the predicted gene models to RNA and protein alignment evidence, and then revises the *ab initio* gene models in order to predict the most confident gene models.

To assess the annotation completeness, the classification strategy adopted for *T*. *aestivum*^47^ was followed. This strategy classified the genes in three main categories, high confidence (HC), low confidence (LC), and transposons (TREP), based on the completeness (start and stop codon) and on the homology (coverage ≥ 90%; e-value ≤ 10 e^−10^) to unipoa (Poaceae proteins, SwissProt and trEMBL), unimag (Magnoliophyta proteins, SwissProt) and TREP database^67^ (transposons database) (Supplementary Table S14).

Finally, InterProScan version 5^68^ was used to classify genes into families and predict the presence of domains and important sites. This software uses 14 different databases, retrieving information such as KEGG^31^ pathways, gene ontology and Pfam domains.

### Synteny Analysis

SyMAP^69^ software was used to search for homologies among *E*. *curvula* and the genomic regions of other grasses. This tool generates whole genome synteny patterns plots between two organisms. The selected species for these comparisons were other C4 grasses, such as *E*. *tef* ^26^ *S*. *bicolor*^24^, *Z*. *mays*^17^, *O*. *sativa*^70^ and *O*. *thomaeum*^14^. SyMAP uses the alignment tool BLAT^71^, run with the following parameters: -minScore = 30, -minIdentity = 70, -tileSize = 10, -qMask = lower, and -maxIntron = 10000.

The paralogous and orthologous syntenic genes obtained were listed (Supplementary Files 1 and 2) and the Ks substitution rate was calculated for each pair of genes using the BioPerl package through the Nei–Gojobori method. To find WGD events the Ks rate was plotted and the peaks were corrected using the method proposed by Wang *et al*.^72^. Results were visualized in a circle plot showing the shared genomic regions with different colors and a table was constructed showing the number of anchors and blocks common to *E*. *curvula*, *E*. *tef*, *S*. *bicolor*, *Z*. *mays*, *O sativa* and *O*. *thomaeum*.

### Analysis of the Evolutionary Relationships Among the Poaceae Family

The evolutionary relationships among *E*. *curvula* and monocots species like *S*. *italica*^23^, *T*. *aestivum*^47^, *E*. *tef* ^8^, *O*. *sativa*^68^, *Z*. *mays*^17^, *S*. *bicolor*^24^, *B*. *distachyon*^46^, *O*. *thomaeum*^14^, *Musa itinerans*^73^ and *P*. *hallii*^74^, were assessed by comparing the assembled genome annotations using the software Orthofinder^75^. This software groups in orthogroups genes originating from the same common ancestor and creates trees for each group and for all the species. The alignment of the orthogroups for all the species was used to create a maximum likelihood time phylogenetic tree using the Jones Taylor Thornton model with the software MEGAX^76^. The calibration was performed according to Prasad *et al*.^77^ considering the divergence time between *O*. *sativa* and *Z*. *mays* in approximately 70 Mya. *M*. *itinierans* was used as outgroup.

Gene expansion and contraction were assessed by the CAFEv3.0^78^ software using the genes families obtained from Orthofinder. Multiple birth-death lambda (λ) was used in order to assess the different clade evolution rates.

### Classification of the WRKY Transcription Factor Family

To identify the WRKYs from *E*. *curvula* the annotated genes with WRKY motifs were extracted from Pfam. The genes were filtered with the MEME software^79^, and those genes showing complete WRKY and zinc finger motifs were classified into three main groups (I, II and III) and into five subgroups (IIa, IIb, IIc, IId and IIe)^48^. Those genes with two WRKY motifs were classified in group I and those with only one into groups II and III. Then, if the terminal region of the zinc finger was H–X_1_–H, the gene was classified as group II and if the terminal region was H–X_1_–C, the gene was classified as group III. Based on the remaining motif of the sequences, group II was classified into five subgroups. Finally, to find the differences among the groups, a multiple sequence alignment was run with the MUSCLE software^80^, all the groups and subgroups were plotted in a phylogenetic tree constructed using MEGAX software^76^ with a maximum likelihood model

### Analysis of Genes Involved in the Lignin Pathway

The KASS online tool^81^ assigns a biological role to new genes using the Ghostx aligner^82^, finding homology to known sequences in the KEGG^31^ database and assigning them a position in a pathway. Genes for class I and class II caffeoyl shikimate esterases (CSE) were targeted, since both have been recently mentioned as having important roles in the regulation of the lignin pathway^32,33^. For BLASTn, a class I *O*. *sativa* orthologous gene (accession XM_015768109.2) and a class II *S*. *bicolor* orthologous gene (accession XM_002462989.2) were used as queries. Specific primers for PCR amplification of these genes were designed based on the genome sequence, the PCR program consisted of an initial DNA denaturation at 94 °C for 2 min, followed by 38 cycles at 94 °C for 15 s, 50 °C for 20 s and 72 °C for 80 s and a final extension of 5 min at 72 °C. Amplicons were analyzed over 1.5% agarose gel. The amplicons were cloned into the pGEM-T Easy Vector (Promega), sequenced and BLAST searched were performed against the *E*. *curvula* transcripts, and the *P halli*, *O*. *sativa*, *E*. *tef*, *O*. *thomaeum*, *Brachypodium distachyon*, *T*. *aestivum*, *Z*. *mays*, *S*. *bicolor* and *S italica* genomes.

### SSRs Discovery and Analysis of Genetic Relationships within the *E*. *curvula* Complex

SSRs discovery was conducted through SSR Locator software^83^ using the assembled genome sequence as input. Aiming to validate the genome assembly and to assess genetic relationships within the *E*. *curvula* complex, primers flanking each SSR were designed using the software Primer3 2.3^84^. Fifteen randomly selected primer pairs (Supplementary Table S9) were synthesized and amplified in genomic DNA from 10 *E*. *curvula* genotypes (Supplementary Table S12). SSRs previously developed for *E*. *curvula* were also tested^9^ to increase the number of markers included in the phylogenetic tree, thus improving the accuracy of the results. PCR reactions used 1 μl of 10 mM dNTPs mix, 1× reaction buffer, 0.5 μl of each forward and reverse primer (100 pmol/μl), 1.5 U Taq DNA polymerase and 60 ng of template genomic DNA in 20 μl of reaction volume. The PCR program consisted of an initial DNA denaturation at 94 °C for 3 min, followed by 40 cycles at 94 °C for 30 s at the optimal annealing temperature for each primer pair and 72 °C for 30 s and a final extension of 5 min at 72 °C. The amplicons were validated in Victoria cultivar using 1.5% agarose gels and the presence/absence of the bands was assessed trough 6% polyacrylamide gels. To size the bands the ladder used was the Genebiotech 100 bp Plus DNA (L00307P).

The similarity of the cultivars and the relative position of cv. Victoria within the *E*. *curvula* complex were assessed regarding the presence or absence of individual bands in the polyacrylamide gels obtained from each primer pair in each individual tested. The pairwise distance of this binary (1 presence, 0 absence) data matrix was computed using the Jaccard method^85^ and the phylogenetic tree was created with hierarchical clustering and the ward D2 method in an R environment.

### Data Access

Under the NCBI bioproject PRJNA508722 (https://www.ncbi.nlm.nih.gov/bioproject/) are available:

-genome sequences

-genome annotation

-DArT reads and markers sequences

## Supplementary information

Supplementary figures and tables

Paralogous genes

Orthologous genes
