# Supplementary material for: A high-quality genome of Eragrostis curvula grass provides insights into Poaceae evolution and supports new strategies to enhance forage quality
Source: Sci Rep. 2019 Jul 15;9:10250. doi: 10.1038/s41598-019-46610-0 (PMC6629639; doi:10.1038/s41598-019-46610-0)
Supplement: Supplementary file 1 — Supplementary figures and tables [file 41598_2019_46610_MOESM1_ESM.pdf]

A high-quality genome of *Eragrostis curvula* grass provides insights into Poaceae evolution and supports new strategies to enhance forage quality

Carballo J, Santos BACM, Zappacosta D, Garbus I, Selva JP, Gallo CA, Díaz A, Albertini E[3], Caccamo M[*] and Echenique V[*]

* Corresponding authors

Echenique V: echeniq@criba.edu.ar

Caccamo M: Mario.Caccamo@niab.com

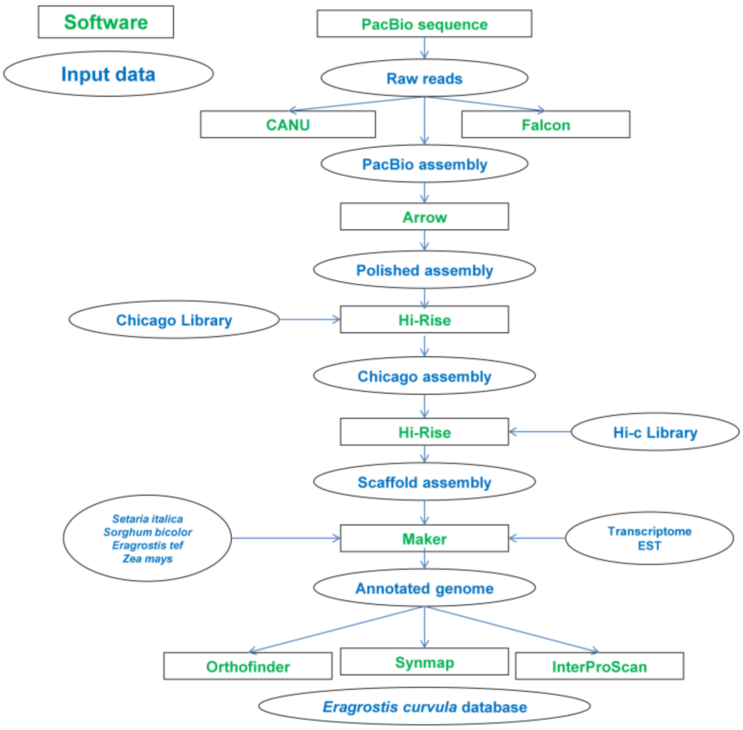

Supplementary Figure S1

Pipeline used to for *de novo* assembly and annotation of *E. curvula* genome. The used software is shown in green rectangles while the input dataset for each program is shown in the blue ovals. The final product was the *E. curvula* genome assembly with the related annotation.

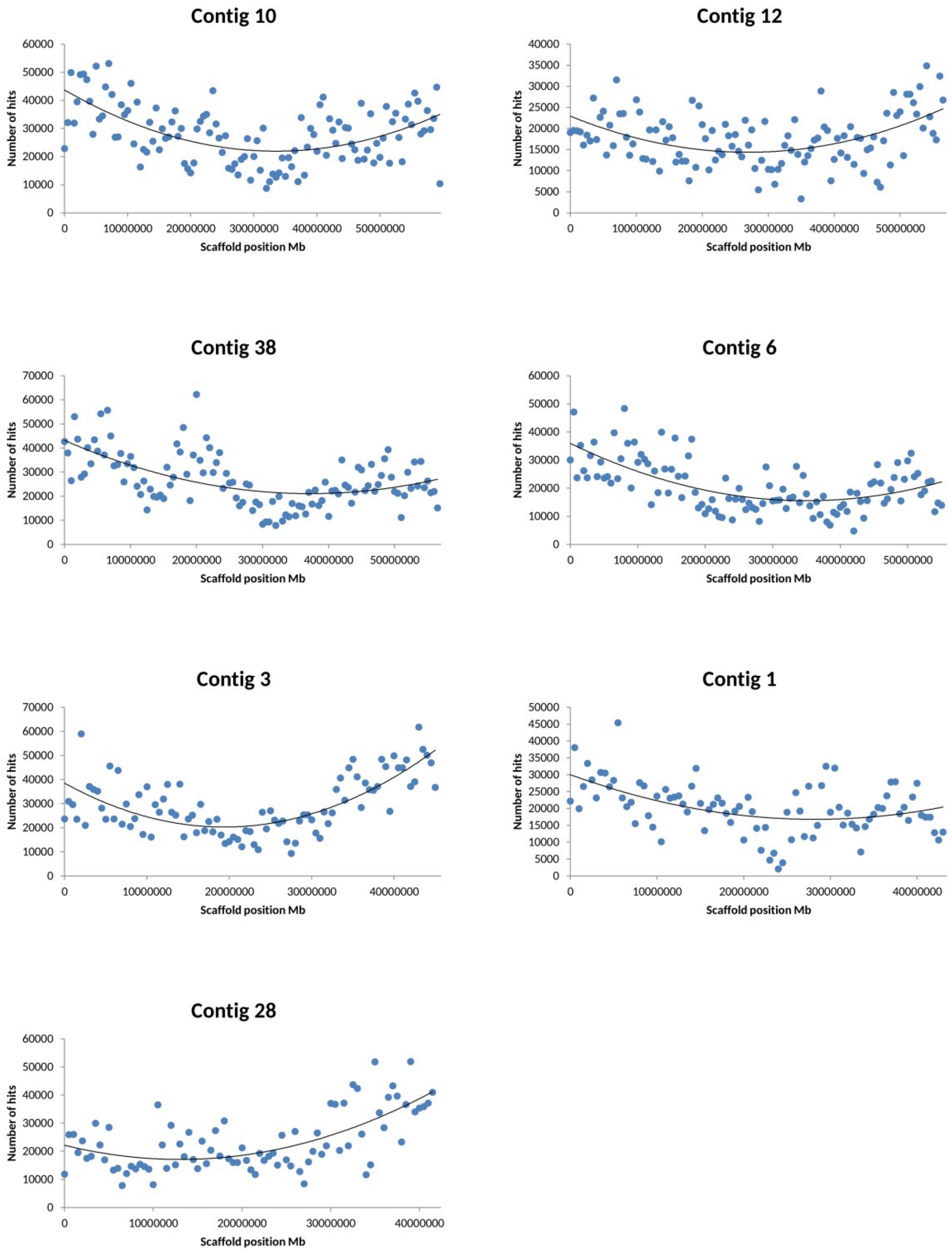

Supplementary Figure S2

Reads from DArT sequencing mapped onto the whole genome assembly. In the figure the seven longest scaffolds are shown and the hits were plotted with a window size of 500 kb.

The number of reads hits is represented in the ordinate whereas the abscissa shows the position on the scaffold in Mb. The curves were obtained applying a quadratic model regression. A positive parabola was obtained with the seven longest scaffolds.

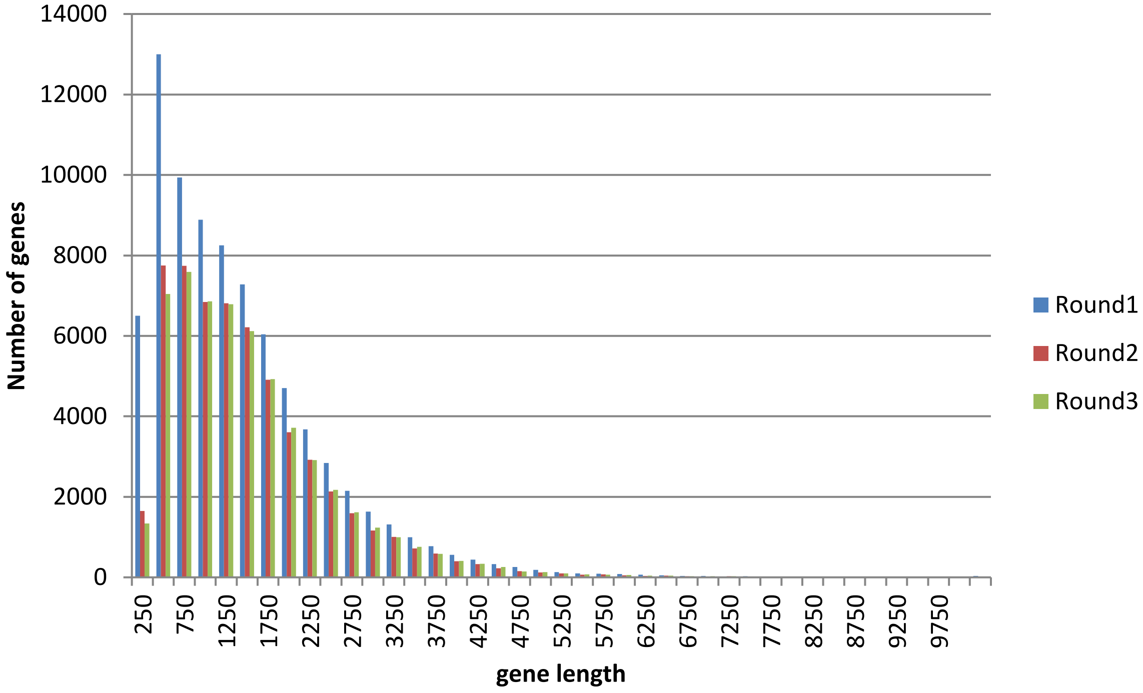

Supplementary Figure S3

Length distribution histograms of gene models after each round of Maker software. The number of genes (ordinate) plotted against the gene length (abscissa), shows the diminution of the number of short length gene model, after each of the three of MAKER software rounds.

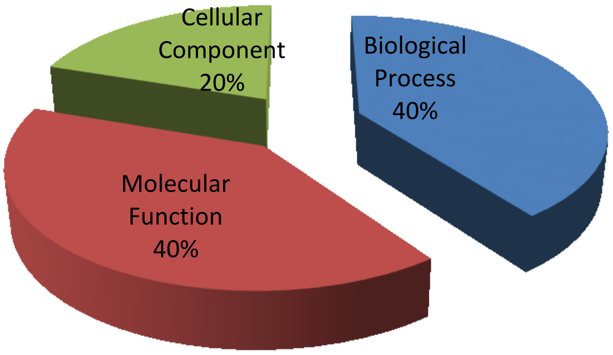

(a)

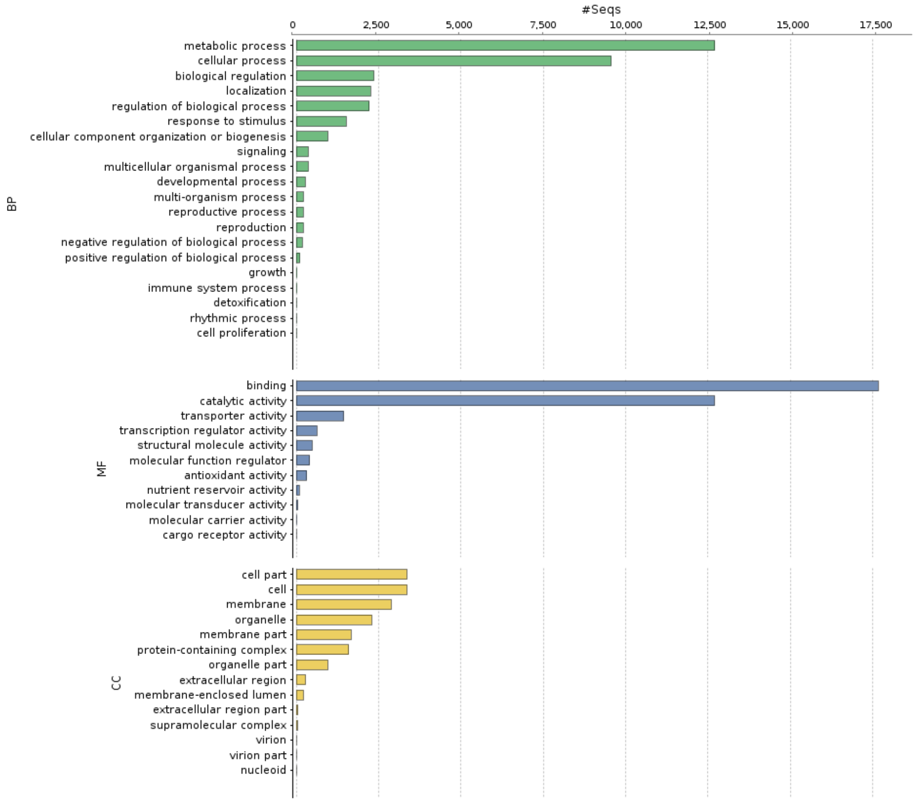

(b)

Supplementary Figure S4.

Functional classification of the whole *Eragrostis curvula* genome representing the distribution of genes based on their annotation in the GO. **a)** Main GO classification: 33,601 genes were classified into biological processes, 17,710 into cellular components and 33,820 into molecular function, finding 29,462 genes with at least one GO annotation. **b)** Distribution of the GO annotation at the second level GO classification. (BP) Biological Process, (MF) Molecular function (CC) cellular component.

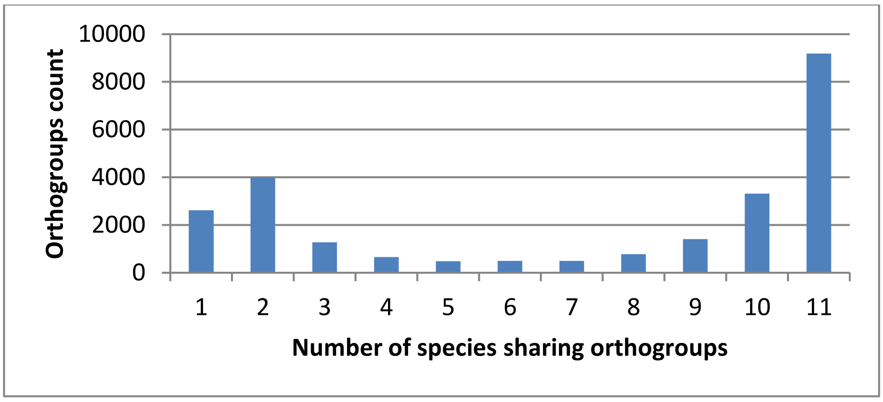

Supplementary Figure S5

Number of grass genomes sharing orthogroups. The genomes considered for this analysis were *E. curvula*, *E. tef* (A and B genomes), *O. thomaeum*, *S. bicolor*, *Z. mays*, *P. hallii*, *S. italica*, *B. distachyon*, *T. aestivum* (A, B and D genomes), *O. sativa* and *M. itinerant.* The number of orthogroups shared by all the species analysed was 9,189 and the second most abundant was 3,991 corresponding to 2 species.

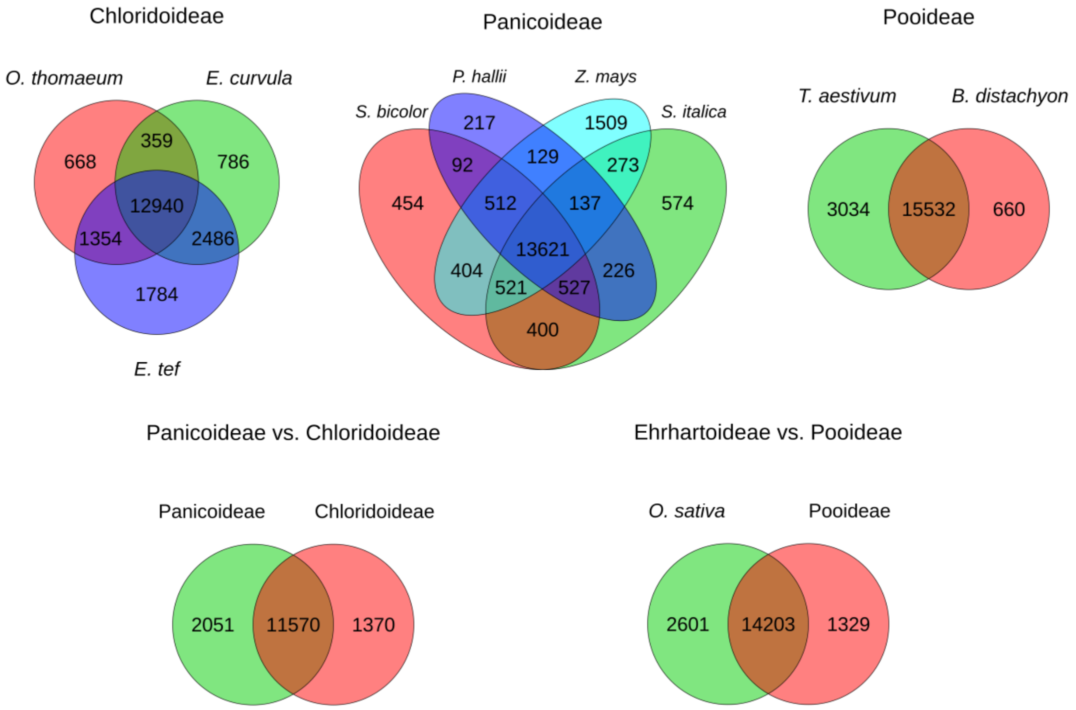

Supplementary Figure S6

The Venn diagrams show the orthogroups shared among the Chloridoideae, Panicoideae, Pooideae and Ehrhartoideae subfamilies of the Poaceae family**.** The Chloridoideae and Panicoideae include all the C4 species selected in this work.

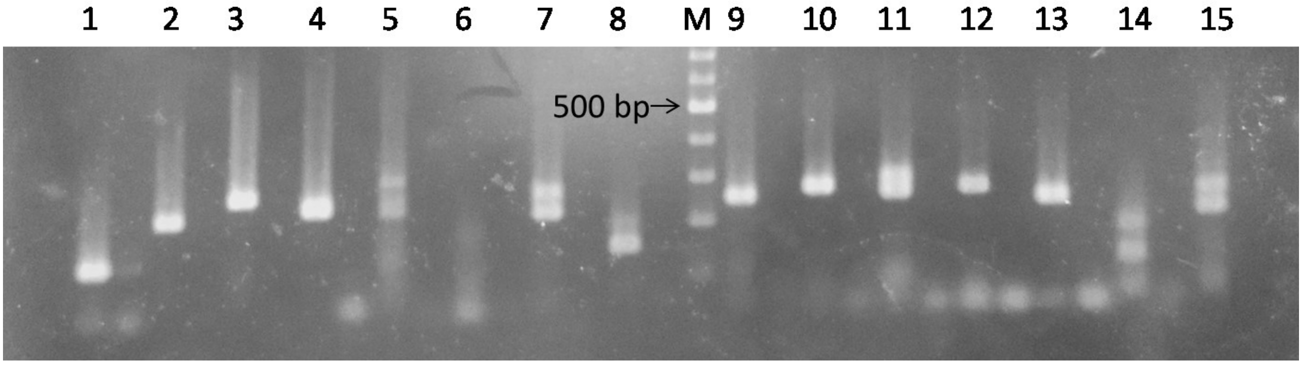

Supplementary Figure S7.

Amplification of SSRs with primers designed based on the cv. Victoria genome assembly. Each number represents a primer pair and between these are the negative controls. M) 100 bp size marker ladder.

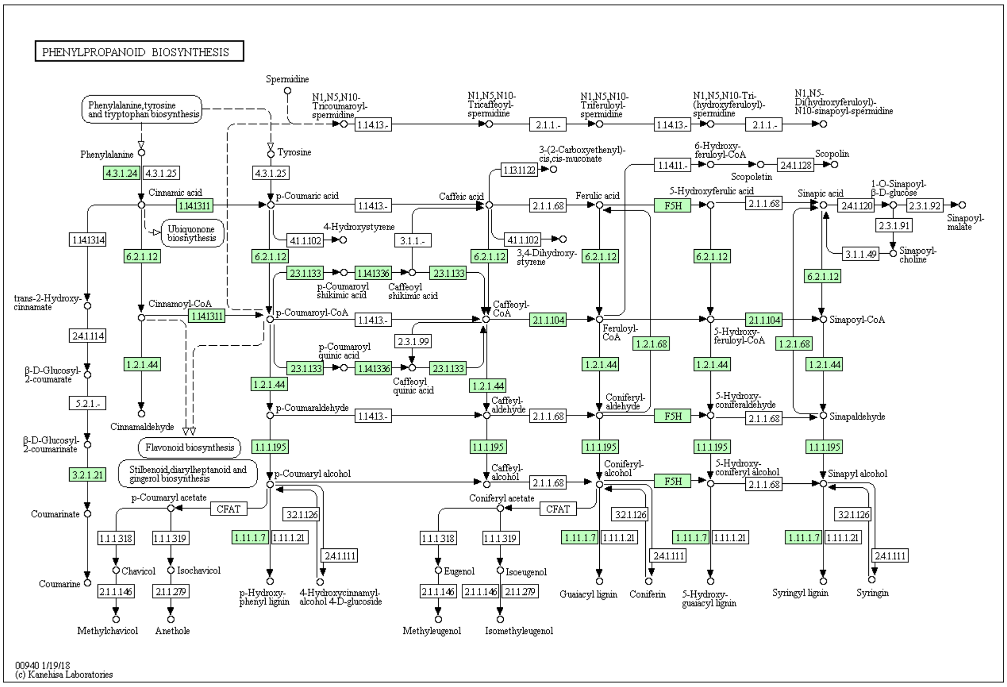

Supplementary Figure S8

Graphic representation of the lignin pathway (map00940) obtained from the KEGG database. The enzymes present in the *E. curvula* gene models are shown in green. In the *E.* curvula gene models was possible to found the whole pathway, from the begging (phenylalanine) at the top left of the figure to the products (guaiacyl, p-hydroxyphenyl, and syringyl) at the botton right of the figure.

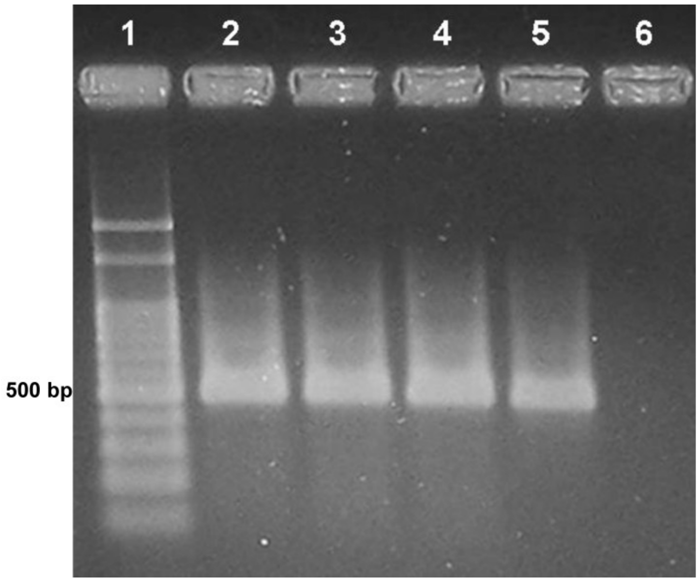

Supplementary Figure S9

E. tef and *E. curvula* CSE class I amplifications. 1) size marker ladder 2) *E. tef* cv. Beten 3) *E. tef* cv. Dabbi 4) *E. tef* cv. Enatite 5) *E. curvula* cv. Victoria 6) Negative control. Equal sized bands represent the presence of the CSE class I in *E. curvula* and *E.tef*. genomes

>TEF-Beten.cv

TCTTCACCCACTCCTTCCACCCGCTCACCGCGTCCCGCGACTGCGAGGTCAAGGCCGTGGGGTTCATGACCCACGGGTAAGGCTCC
GACACCTAGTGGTTGTTCCAGTTGATGGCCATCAGCTACGCGAGGTGGGGTACGCCGTCTTCTGCTCGGACCTCCTCGGCCACGG
CCGATCCGACGGTGTCCACGGCTACCTCGGCGACATGGAGGCCACGGCCGCCGCGTCGCTCGCCTTATTCCTCTCCGTGCGGCCGA
GCGAGCCATACGCGCAGCTCCCGGCGTTCCTTTTCGGCGAGTCCATGGGGGGAGCCGCCACCCCGGTCATGGACCTCCGGTCCCC
GTAGCTCGACGCCGAGGGGACGGGGCTCATCTTCTCCGCGCCGCTCTTCGTGATCCCGGAACACATGTACCCGTCCCGGATTCGCC
TGTTCCTGTCCGAACACCACGTCCGCCTCGTCGACATGTGGGCGGTGCTGCCGGACAAGAAGAGGGTGGGCAAGTC

>TEF-Dabbi.cv

TCTTCACCCACTCCTTCCACCCGCTCACCGCGTCCCGCGACGGCGACGTCAAGGCCGTGGTGTTCATGACCCACGGCTACGGCTCC
GACACCTCGTGGCTGTTCCAGTCGATCGCCATCAGCTACGCGCGCTGGGGTACGCCGTCTTCTGCGCGGACCTCCTCGGCCACGG
CCGCTCCGACGGTGTCCACGGCTACCTCGGCGACATGGAGGCCACGGCCGCCGCGTCGCTCGCCTTCTTCCTCTCCGTGCGGACGA
GCGAGCCATACGCGCAGCTCCCGGCGTTCCTCTTCGGCGAGTCCATGGGCGGCGCCGCCACGCTGCTCATGTACCTCCGGTCCCCG
TCGCGCGACGCCGCGTGGACGGGGCTCATCTTCTCCGCGCCGCTCTTCGTGATCCCGGATGACATGTACCCGTCCCGGATTCGCCT
GTTCCTGTACGGCCTCCTCGTCGGCCTCGCCGACACGTGGGCGGTGCTCCCGGACAAGAAGATGGTGGGCAAGTC

>TEF-Enatite.cv

TCTTCACCCACTCCTTCCACCCGCTCTCCGCCGCGTCCAGCGACGGCGACGTCAAGGGCGTGGTGTTCATGACCCACGGCTACGGC
TCCGACACATCGTGGCTGTTCCAGTCCATCGCCATCAGCTACGCGCGCTGGGGTACGCCGTCTTCTGCGCGGACCTCCTCGGCCA
CGGCCGCTCCGACGGCGTCCACGGCTACCTCGGCGACATGGAGGCCGCGGCCGCCGCGTCGCTCGCCTTCTTCCTCTCCGTGCGG
ACGAGCGAGCCCTACGCGCGCCTCCCGGCGTTCCTCTTCGGCGAGTCCATGGGCGGCGCAGCGACGCTGCTCATGTACCTCCGGTC
CCCGTCGCGTGACGCGGCGTGGACGGGGCTCATCTTCTCCGCGCCGCTCTTCGTGATCCCCGACGACATGTACCCGTCCCGTGTGC
GGCTCTTCCTTTACGGCCTCCTCTTCGGGCTCGCCGACACGTGGGCCGTGCTCCCGGAAAAGAAGATGGTGGGCAAGTC

Supplementary Figure S10.

Sequences obtained from the amplified clones of the CSE class I on the *E. tef* cultivars Betem, Dabbi an Enatite. The sequences confirm the presence in this species.

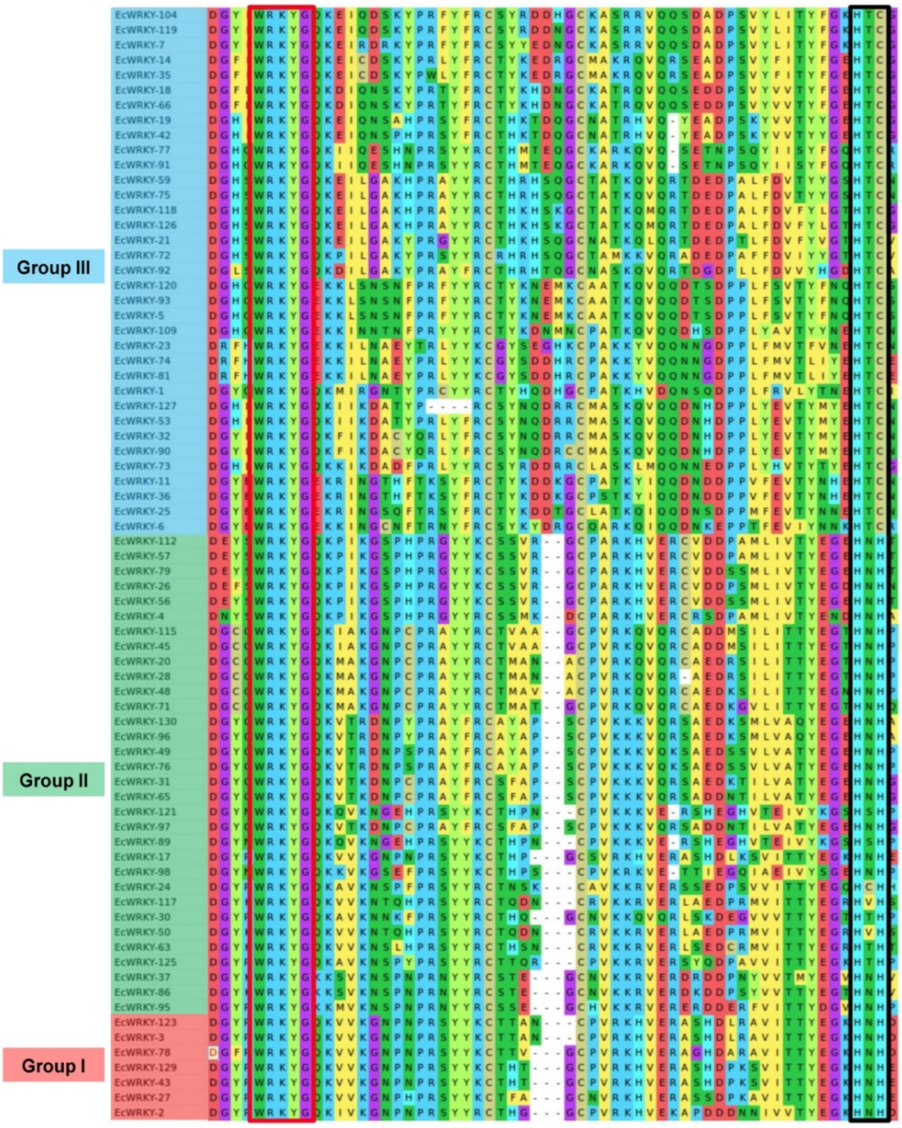

Supplementary Figure S11

Pairwise alignment of the protein sequence containing the WRKY domains. The WRKY motifs are highlighted in a red frame and the Zinc finger structures are shown in a black frame. In Group III (light blue) the Zinc finger motif tail amino acids are HTC, while in Group I (red) and Group II (green) they are Hx_1H.

Supplementary Table S1

Length (bp), number of genes and number of DArT markers on the 14 longest contigs.

Name	Length	Genes	DArT
**Contig10**	59,696,155	6,315	694
**Contig12**	57,091,500	4,450	489
**Contig38**	57,060,119	5,759	623
**Contig6**	55,809,479	5,036	550
**Contig3**	45,712,337	4,943	440
**Contig1**	43,444,889	3,164	278
**Contig28**	42,455,315	3,888	397
**Contig30**	29,802,391	3,198	447
**Contig8**	28,838,055	3,393	326
**Contig50**	26,185,977	2,345	247
**Contig25**	23,821,556	2,503	286
**Contig4**	21,583,994	1,943	249
**Contig44**	8,977,321	705	75
**Contig33**	4,567,900	320	25

Supplementary Table S2

Length (in bp) of the E. curvula genome covered by repetitive elements, classified using the software RepeatClassifier. The number of bases covered by repetitive elements in the main group is higher than the sum of the classes because the residual redundancy in the TE/repeat library causes RepeatMasker to output overlapping intervals

Repetition Type	Length covered bp		
	Main group	Class	Subclass
**DNA**	24,353,008		
**DNA/CMC-EnSpm**		8,443,508	
**DNA/Dada**		380	
**DNA/hAT**		3,602,100	
**DNA/hAT-Ac**		2,299,433	
**DNA/hAT-Blackjack**		220	
**DNA/hAT-Tag1**		612,449	
**DNA/hAT-Tip100**		681,906	
**DNA/MuLE-MuDR**		3,519,839	
**DNA/Novosib**		21,382	
**DNA/PIF**		3,680,469	
**DNA/PIF-Harbinger**			3,680,402
**DNA/Sola-2**		174	
**DNA/TcMar-Stowaway**		1,108,579	
**LINE/I**		112,095	
**LINE/I-Jockey**			39,160
**LINE/L1**		8,791,643	
**LINE/L1-Tx1**			101,619
**LINE/L2**		156,955	
**LINE/Penelope**		178,991	
**LINE/R1**		19,915	
**LINE/RTE-BovB**		118,146	
**LINE/RTE-X**		732	
**Low_complexity**	754,861		
**LTR**	103,420,290		
**LTR/Cassandra**		250,669	
**LTR/Caulimovirus**		497,433	
**LTR/Copia**		19,200,873	
**LTR/Gypsy**		83,036,168	
**LTR/Lenti**		2,991	
**LTR/Ngaro**		135	
**LTR/Pao**		1,395	
**RC/Helitron**		2,667,299	
**Retroposon/L1**		50,806	
**rRNA**	212,362		
**Satellite**	6,964		
**Satellite/centr**		763	
**Simple_repeat**	7,930,578		
**SINE**	845,966		
**SINE/ID**		3,232	
**SINE/L1**		103	
**SINE/tRNA**		815,240	

Repetition Type	Length covered bp		
	Main group	Class	Subclass
SINE/tRNA-L1		1,858	
SINE/tRNA-RTE		21,943	
snRNA	6,886		
tRNA	834,401		
Unknown	27,627,765		

Supplementary Table S3.
Gypsy and *Copia* LTR elements ratio and their percentage in the principal C_4 Poaceae species.

	E. curvula	*E. tef*	*S. bicolor*	*S. italica*	*Z. mays*
***Copia* %**	3.14	2.67	5.18	7.18	18.3
***Gypsy* %**	13.62	11.4	19	22.14	34.88
**Ratio**	4.34	4.27	3.67	3.08	1.91

Supplementary Table S4

Repeats reported in previous work related to EST EH191456.1. The *Gypsy* repetitive element was formerly found by homology with the *Oryza sativa* AAP53838 accession.

EH191456.1 alignment				Repeat position			
**Contig**	**start**	**end**	**length**	**Contig**	**Repeat start**	**Repeat end**	**ID**
Contig10	38,306,648	38,307,286	638	Contig10	38,304,264	38,308,883	Target=gypsy.56-1440.Contig10.38304691-38308737 1 4628;ID=31226
Contig1	18,815,676	18,816,298	622	Contig1	18,814,081	18,818,681	Target=gypsy.112-1440.Contig1.18814338-18818256 1 4626;ID=6809
Contig1	21,539,544	21,540,157	613	Contig1	21,537,625	21,541,508	Target=gypsy.112-1437.Contig1.21537625-21541508 1 3884;ID=7663
Contig12	37,772,330	37,,772,937	607	Contig12	37,765,412	37,788,789	Target=rnd-6_family-2111 1 13557;ID=57119
Contig12	37,782,180	37,782787	607	Contig12	37,765,412	37,788,789	Target=rnd-6_family-2111 1 13557;ID=57119
Contig251	31,145	31,784	639	Contig251	28,768	32,337	Target=gypsy.56-1440.Contig251.29194-33231 871 4628;ID=78046
Contig25	18,621,160	18,621,775	615	Contig25	18,615,195	18,628,614	Target=rnd-6_family-2111 519 10333;ID=76061
Contig28	2,317,209	2,317,848	639	Contig28	2,315,739	2,320,231	Target=gypsy.112-1440.Contig28.2315874-2319805 1 4628;ID=80106
Contig28	26,402,399	26,403,012	613	Contig28	26,401,991	26,404,363	Target=gypsy.112-922.Contig28.26401991-26404363 1 2373;ID=89340
Contig30	10,585,659	10,586,298	639	Contig30	10,585,106	10,587,942	Target=gypsy.56-1440.Contig10.38304691-38308737 884 3732;ID=118269
Contig30	25,223,532	25,224,170	638	Contig30	25,222,080	25,226,129	Target=gypsy.56-1440.Contig449.81940-85989 1 4050;ID=123558
Contig3	3,265,371	3,266,008	637	Contig3	3,263,755	3,268,700	Target=gypsy.114-1440.Contig3.3264039-3267968 1 3930;ID=97756
Contig34	1,121,031	1,121,668	637	Contig34	1,119,576	1,123,628	Target=gypsy.56-1440.Contig34.1119576-1123628 1 4053;ID=128837
Contig38	44,894,105	44,894,743	638	Contig38	44,893,549	44,897,127	Target=gypsy.56-1440.Contig10.38304691-38308737 884 4627;ID=148682
Contig393	273	909	636	Contig393	1	3,717	Target=gypsy.471-1440.Contig393.1-2866 1 4628;ID=153306
Contig449	83,899	84536	637	Contig449	81,164	85,989	Target=gypsy.56-1440.Contig449.81940-85989 1 4050;ID=166244
Contig6	35,490,116	35,490,723	607	Contig6	35,483,202	35,496,727	Target=rnd-6_family-2111 1 13554;ID=195802
Contig6	47,488,703	47,489,343	640	Contig6	47,486,746	47,490,685	Target=gypsy.112-1440.Contig6.47486746-47490685 1 3940;ID=200341
Contig6	47,633,185	47,633,825	640	Contig6	47,631,204	47,634,379	Target=gypsy.112-1440.Contig6.47631204-47635173 768 3970;ID=200381
Contig6	55,164,880	55,165,519	639	Contig6	5,516,2187	55,166,988	Target=gypsy.56-1440.Contig10.38304691-38308737 1 4047;ID=203015

EH191456.1 alignment				Repeat position			
**Contig**	**start**	**end**	**length**	**Contig**	**Repeat start**	**Repeat end**	**ID**
Contig708	22,751	23,390	639	Contig708	21,108	23,944	Target=gypsy.56-1440.Contig10.38304691-38308737 885 3733;ID=207612
Contig853	202,818	203,455	637	Contig853	200,859	204,908	Target=gypsy.56-1440.Contig853.200859-204908 1 4050;ID=224234
Contig947	19,017	19,652	635	Contig947	16,633	21,264	Target=gypsy.56-1440.Contig947.17059-21105 1 4628;ID=228637

Supplementary Table S5

Gene annotation. Average length, number of genes and BUSCO results after each round of the MAKER software.

	Average length bp	genes	BUSCO
**Round 1**	1,303.8	80,481	C:89.0%[S:51.1%,D:37.9%],F:5.1%,M:5.9%,n:1,440
**Round 2**	1,392.9	57,395	C:93.2%[S:66.5%,D:26.7%],F:1.9%,M:4.9%,n:1,440
**Round 3**	1,424.03	56,469	C:93.4%[S:65.3%,D:28.1%],F:1.7%,M:4.9%,n:1,440

Supplementary Table S6

E. curvula protein matches in the InterProScan and TrEMBL databases. The TrEMBL database was filtered for more than 75 % of identity and 90 % of coverage.

**InterProScan databases**	
**Database**	**Number of matches**
SFLD	292
ProDom	539
Hamap	1008
PIRSF	2,215
TIGRFAM	3,353
PRINTS	5,834
Coils	6,971
ProSitePatterns	7,657
SMART	12,257
CDD	13,001
ProSiteProfiles	15,495
MobiDBLite	26,335
SUPERFAMILY	29,770
Gene3D	29,980
Pfam	35,713
PANTHER	44,532
Kegg	10,005
GO	29,429
**TrEMBL database**	
TrEMBL	18,955

Supplementary Table S7.

Synteny relationships between E. curvula and Z. mays, S. bicolor, O. thomaeum and E. tef. The coverage is the percentage of the genome length covered by syntenic blocks. Species coverage indicates the coverage of the E. curvula genome over the genome of the species in the first column.

Species	Blocks	anchors	*E. curvula* coverage	Species coverage	Inverted
Z. mays	363	42,818	79%	94%	182
S. bicolor	305	44,051	79%	66%	149
O. thomaeum	251	35,121	85%	96%	124
O. sativa	262	36,698	84%	98%	131
E. tef	138	40,370	85%	98%	66

Supplementary Table S8

SSRs primers tested with different E. curvula genotypes. The primers indicated with "*" are primers designed based on the reference transcriptome of the species (Garbus et. al 2017) and the primers starting with "Victoria" were designed based on cv. Victoria genome. The predicted amplicons size was measure mapping the primers to the *E. curvula* genome and the obtained amplicons size was determinate using a ladder in an agarosa gel.

Primer Name	Primer Forward	Primer Reverse	SSR Motif	Aligned Contig	Amplicon in Victoria (bp)		% Identity		% Coverage	
					Predicted	Obtained	Primer Forward	Primer Reverse	Primer Forward	Primer Reverse
Victoria-N1	GTCCCTGCAGTCAGAGTAGC	ACGAAGGGGAGGATGAAAGC	(CTG)5	Contig10	145	140	100	100	100	100
Victoria-N2	TTCCTTTGTTGTGGGGCCAT	TGAGTATCCACACGCAGCTG	(TGT)5	Contig10	235	240	100	100	100	100
Victoria-N3	TGCTATTGGACGGTGCAGTT	GAGCAATTCGGAGGGGAGAG	(TGT)5	Contig30	276	270	100	100	100	100
Victoria-N4	ATCCCTACGTCAACGATGGC	GGCTTTTGGCACTCGTCAAT	(TCT)12	Contig30	242	260	100	100	100	100
Victoria-N5	CCCATCTCCAAGACCAAGCA	TGCCGTAGTAGGGAGAAGGA	(CCTTT)5	Contig30	263	260	100	100	100	100
Victoria-N6	CCACACTGAAGACAAGACAGC	ACCCTGGGTGTGAATTAGCG	(GT)9(AT)31	Contig936	142	Unspecific	100	100	100	100
Victoria-N7	AGCATGGTCGAACGGTGAAT	AACTGAGGATGCCTAGCTGC	(CTGG)5	Contig38	227	230	100	100	100	100
Victoria-N8	GTCACAGCGCCAAAGAAAGG	TGGGATCAGGATGGGTAGGG	(AGAC)5	Contig38	159	150	100	100	100	100
Victoria-N9	CGTGAAAGAGAAGGCGCAAC	CAGCAGCGGTGAGAAGAAGA	(GCCT)5	Contig28	251	250	100	100	100	100
Victoria-N10	CTTCACCAAGCTCCGGAAGT	ACCCCCTAAGAGATCCAGCA	(TTTCCT)5	Contig28	270	270	100	100	100	100
Victoria-N11	GCATGTTGACCTCCACTCCA	ATCTCAGTGACCCATGTGCC	(TTTTC)6	Contig8	260	260	100	100	100	100
Victoria-N12	CTCGAGCTAGAGCCAGATGC	TGGAATCGGCATCGGTGTAG	(GAGCCG)5	Contig38	270	270	100	100	100	100
Victoria-N13	GCCAACCCCAGCACTCTTAT	GGAGAGGTGGAAAAGGTGGG	(ATCTCT)5	Contig3	244	240	100	100	100	100
Victoria-N14	GGTGGTGGGGGTGAGTAAAG	AGAGTCAAAAGCGTTCCCGT	(GGCAC)5	Contig190	136	No amplification	100	100	100	100
Victoria-N15	AGACACCATCTCCAACAGCG	GTTCCCCTAAGTCACGGCAA	(GCTCG)5	Contig4	218	230	100	100	100	100
isotig01946*	TCCATATCCATCTACGAACC	GTTCGTTGTGAATTTTTGCT	(TCT)5		Not found	No amplification	-	-	-	-

isotig06745*	GTGAAGGAGGAGAAGTCGAG	GGAGAGCGAGTCGTCC	(CCG)5	Contig30	291	300	100	100	100	100
isotig07764*	CCTGCTTCTTGCTCCC	AAAGAAGACGTCGTAAGCAG	(CCG)6	Contig1	186	170	100	100	100	85
isotig09593*	TGACATGTGACAGACTCCAT	CGTATATTGGCATCTCGTCT	(T)108		Not found	No amplification	-	-	-	-
isotig10159*	TAGCCAGATGACCTCCAC	CCTGCTCTCCTCCGAC	(GGA)8	Contig50	244	310	100	100	100	100
isotig13242*	GCTCGACTCCGTCTCC	GAAACCGTCCAAGAAGAAG	(CTT)5	Contig25	187	No amplification	100	100	100	100
isotig13665*	TGCGCTGGACCTCTACTA	ACTGCTTCAGCCTCATCTT	(CGG)6	Contig25	352	300	100	100	94.44	100
isotig13699*	GTTCAGCATGAGTAACCACA	CTCCTTCCTCTCCTCATTCT	(AGAAGG)6	Contig12	321	300	100	100	100	95
isotig13776*	TCCAACTCATCAACCAGTAA	GTAGCTCTTGCCGAACC	(CCT)5	Contig33, Contig175	227	230	100	100	100	100
isotig15838*	AGTGGAGGAAGATGTAGCC	ACAGAGTTGAAGGAGCAGAG	(C)10	Contig50, Contig12, Contig6	241	250	100	100	100	100
isotig18079*	GACAGGACCCCTCTTCC	AGGACTCCCAGCTCAGAT	(GGC)5	Contig705	153	150	100	100	100	100
isotig18488*	ACAGAAACCAACAGTGGAAC	GATGGTTCCGTAGATCGTAG	(CA)6	Contig685	233	260	100	100	100	100
isotig20053*	CCACCAACCAATTATCCTAA	GATCTCGCGACAACCC	(CAC)7	Contig38	236	250	100	100	100	100
isotig20164*	GAACTACCTGCAGATCAAGG	TGCACTTTGTTGTCATCTTC	(GAT)7	Contig38	184	200	100	100	100	100
isotig23517*	CTGCATCCAGTGGTTTTC	ATGTACTCAAGGCACGACA	(GCC)6	Contig50	171	160	100	100	100	100
isotig23773*	ACCGCATTCTTTGATTTAGA	TGTGATTTCTTCGGTTCTTT	(GAG)5	Contig25	211	200	100	100	100	100
isotig24162*	TCATCTTCCTCTTGATCTGC	ATGGAAGCAGAGCACTACC	(TCG)5		Not found	No amplification	-	-	-	-
isotig25156*	ACCACAGTACGGACACTCTC	CGTACTCCTTGTAACGCTG	(GAG)6	Contig28	771	800	100	100	100	100
isotig25372*	GATGACAACCACAATAAGCC	ATTTCTTCCACGACGAAGTT	(CCG)5	Contig50	163	160	100	100	100	100
isotig25706*	GGTGAGGAAGTCGAGGAT	ACTTCAAGGTGGCGTACA	(GTT)8	Contig28	245	Unspecific	100	100	100	100
isotig27380*	GTTCGGCGTCTCCATC	GTATATGGGCCAGTGGTTC	(TCGCCG)5	Contig38	193	200	100	100	100	100
isotig27968*	ACTTGCAGAAATCACAAAGG	GGATCGTGTTGATTGAAGTT	(A)12	Contig38	158	160	100	100	100	100
isotig28255*	GTCGTTGAACATGATCCCT	GAGCAGGAGAACATCAAGAG	(CGC)5	Contig30	340	300	100	100	100	100
isotig28834*	GCGTAGCTCTTCTCCATGA	CACTTCTTCGGGGTGTTC	(CGG)5	Contig188, Contig3	306	300	100	100	100	100
isotig29328*	AGCATCATCACCAATTCCT	GTAACGTAGGGTGGGAATC	(GCA)5	Contig30	370	No amplification	100	100	73.68	100

isotig30246*	TCTTCTTCAGCCCCTTG	ACGTCGTCGTCATCCTC	(GTC)5	Contig3, Contig10	113-249	160	100	100	100	100
isotig32396*	CGCCATGAAGCATCTC	CAACTTTGTTTTTCCTCGTC	(GCC)5		Not found	No amplification	-	-	-	-
isotig33661*	ATGGCATCTTCTCCTGTGAT	GAACCCCTTCGCAGC	(CGC)5	Contig12	152	150	100	100	100	100
isotig33732*	ATCCTGTTTTTGTGCTTGTT	GGGAAAAAGCTCCAGTAATC	(CCT)7		Not found	No amplification	-	-	-	-
isotig33734*	GTCCATCCGATCCCAG	AACTCATCTTTGCACGACTT	(CGC)5	Contig3	666	250	100	100	100	100
isotig34134*	TACTCATCGTCGTACCTCCT	GGCTGAGTACTTGGTCTCTC	(GCG)5	Contig10	168	170	100	100	100	100
isotig35118*	CATCATCATCCCCATTTATC	TGATCACAAGATGACCAAGA	(GAA)5	Contig1	284	300	100	100	100	95
isotig35144*	ACAAGTCATCGGACTCCTC	AGGGAGAGAGACGAGAAACT	(TCC)5		Not found	300	-	-	-	-
isotig38544*	AAAAAGCGGAAGCAGAAC	CGCTTGAATCAGCATAGG	(GGC)7	Contig155, Contig902	244	No amplification	100	100	100	100
isotig42614*	GATTCAACCGTACTTCAGGA	AAAAACCTCATCTCCCCC	(AG)7		Not found	No amplification	-	-	-	-

Supplementary Table S9

A) Primers designed to specifically amplify class I and II CSE genes from *E. curvula*. The amplicon length was estimated from the genome sequence comprised between F and R primers and the coding region is from the start to the stop codon.

Primer name	Primer forward	Primer reverse	Aligned contig	Forward start	Reverse end	Amplicon	Coding
***E.curvula*-CSE-Class-I**	GAACATCGACTGCACCGAAC	AGGGGCACTGTGCTGATTTA	Contig10	52,957,431	52,956,315	1,116	1,014
***E.curvula*-CSE-Class-II**	AACCCAAGTCTGATCCCCCG	GATAGACTTGTTGGTCAGGTTTAC	Contig6	10,758,145	10,759,319	1,174	1,023
***E.tef*-CSE-Class-I**	TCTTCACCCACTCCTTCCA	GACTTGCCCACCATCTTCTT	Contig10	52,957,233	52,956,732	501	

Supplementary Table S10

CSE I and II gene sequences cloned from genomic DNA of *E. curvula* aligned against the sequenced genome of the species (column one). % Ident = identity, % Cov = coverage, Mm = mismatch

Species	Query ID	Target ID	% Ident	% Cov	Length	Mm	Gap	Query start	Query end	Target start	Target end	E-val
E curvula												
	E. curvula Class II	Contig6	100	100	1014	0	0	1	1,014	10,797,008	10,798,021	0
	E. curvula Class I	Contig10	100	100	1023	0	0	1	1,023	52,957,363	52,956,341	0
***E. curvula* transcriptome**												
	E. curvula Class II	isotig39171	98.915	100	1,014	11	0	1	1,014	1,079	66	0
	E. curvula Class I	isotig40238	96.752	84	862	20	6	165	1,023	1,023	167	0
O. thomaeum												
	E. curvula Class II	gi|7	88.889	100	1,017	100	4	1	1,014	4,574,595	4,575,601	0
	E. curvula Class I	gi|4	88.583	96	762	80	4	223	977	4,063,536	4,064,297	0
	E. curvula Class I	gi|4	87.946	96	224	27	0	1	224	4,058,612	4,058,835	1.98E-74
E. tef												
	E. curvula Class II	CL3332Contig4	94.655	100	1,029	37	5	1	1,014	210	1,235	0
P. halli												
	E. curvula Class II	ref|NC_038042.1|	88.287	100	1,033	98	6	1	1,023	5,724,226	5,723,207	0
	E. curvula Class I	ref|NC_038043.1|	91.735	96	980	66	3	37	1,013	52,404,597	52,403,630	0
O. sativa												
	E. curvula Class II	2	87.087	95	968	125	0	19	986	6,039,452	6,,038,485	0
	E. curvula Class I	7	87.29	93	952	98	7	52	999	22,694,322	22,693,390	0
**Class II only species**												
Z. mays												

Species	Query ID	Target ID	% Ident	% Cov	Length	Mm	Gap	Query start	Query end	Target start	Target end	E-val
	E. curvula Class II	2	89.98	96	978	80	11	26	998	212,556,872	212,555,908	0
S. bicolor												
	E. curvula Class II	gb|CM000761.3|	90.419	98	1,002	75	11	21	1,013	70,832,179	70,831,190	0
S. italica												
	E. curvula Class II	II	91.179	96	975	71	7	26	997	43,454,946	43,453,984	0
B. distachyon												
	E. curvula Class II	1	87.187	98	999	106	9	19	1,007	19,776,425	19,777,411	0
T. aestivum												
	E. curvula Class II	2D	86.735	95	980	109	5	47	1,014	163,020,312	163,019,342	0
	E. curvula Class II	2A	85.944	97	996	113	6	34	1,014	176670726	176,671,709	0
	E. curvula Class II	2B	86.27	95	976	110	6	47	1,007	224,649,275	224,650,241	0

.

Supplementary Table S11

WRKY gene positions on the *E. curvula* contigs. Groups with the same number represent WRKY genes separated less than 100 kb from each other

Name	Contig	Start	End	Distance	Group
**EcWRKY-57**	Contig1	7,639,664	7,640,664	0	
**EcWRKY-112**	Contig1	7,816,970	7,817,970	176,306	
**EcWRKY-27**	Contig1	17330673	17331673	9,512,703	
**EcWRKY-25**	Contig1	26,783,469	26,784,469	9,451,796	1
**EcWRKY-5**	Contig1	26,810,270	26,811,270	25,801	1
**EcWRKY-65**	Contig10	51,,394,540	51,395,540	0	
**EcWRKY-30**	Contig12	30,081,135	30,,082,135	0	
**EcWRKY-6**	Contig12	37,747,966	37,748,966	7,665,831	
**EcWRKY-120**	Contig12	54,776,336	54,777,336	17,027,370	2
**EcWRKY-36**	Contig12	54,783,316	54,784,316	5,980	2
**EcWRKY-11**	Contig12	55,014,645	55,015,645	230,329	3
**EcWRKY-93**	Contig12	55,019,375	55,020,375	3,730	3
**EcWRKY-48**	Contig25	7,31,727	7,432,727	0	
**EcWRKY-117**	Contig25	92,654,11	9,266,411	1,832,684	
**EcWRKY-28**	Contig25	23,499,454	23,500,454	14,233,043	
**EcWRKY-71**	Contig28	12,535,306	12,536,306	0	
**EcWRKY-18**	Contig28	35,399,156	35,400,156	22,862,850	4
**EcWRKY-81**	Contig28	35,416,567	35,417,567	16,411	4
**EcWRKY-119**	Contig28	35,426,181	35,427,181	8,614	4
**EcWRKY-66**	Contig28	35,452,888	354,53,888	25,707	4
**EcWRKY-74**	Contig28	35,465,844	35,466,844	11,956	4
**EcWRKY-104**	Contig28	35,475,743	35,476,743	8,899	4
**EcWRKY-7**	Contig28	35,504,971	35,505,971	28,228	4
**EcWRKY-23**	Contig28	35,524,890	35,525,890	18,919	4
**EcWRKY-35**	Contig28	35,534,799	35,535,799	8,909	4
**EcWRKY-14**	Contig28	36,330,994	36,331,994	795,195	
**EcWRKY-86**	Contig28	40,538,190	40,539,190	4,206,196	
**EcWRKY-4**	Contig3	4,817,141	4,818,141	0	
**EcWRKY-26**	Contig3	9,138,417	9,139,417	4,320,276	
**EcWRKY-79**	Contig3	19,118,216	19,119,216	9,978,799	
**EcWRKY-42**	Contig3	32,906,754	32,907,754	13,787,538	
**EcWRKY-19**	Contig3	33,078,890	33,079,890	171,136	
**EcWRKY-109**	Contig3	33,675,832	33,676,832	595,942	
**EcWRKY-32**	Contig30	7,853,877	7,854,877	0	5
**EcWRKY-127**	Contig30	7,885,213	7,886,213	30,336	5
**EcWRKY-63**	Contig30	15,205,357	15,206,357	7,319,144	
**EcWRKY-31**	Contig38	6,380,798	6,381,798	0	
**EcWRKY-21**	Contig38	14,591,241	14,592,241	8,209,443	6
**EcWRKY-59**	Contig38	1,4598,763	14,599,763	6,522	6

Name	Contig	Start	End	Distance	Group
**EcWRKY-72**	Contig38	14,607,427	14,608,427	7,664	6
**EcWRKY-126**	Contig38	14,617,706	14,618,706	9,279	6
**EcWRKY-49**	Contig38	17,714,206	17,715,206	3,095,500	7
**EcWRKY-130**	Contig38	17,722,243	17,723,243	7,037	7
**EcWRKY-76**	Contig38	17,774,932	17,775,932	51,689	7
**EcWRKY-96**	Contig38	17,782,957	177,83,957	7,025	7
**EcWRKY-17**	Contig38	21,862,854	21,863,854	4,078,897	
**EcWRKY-45**	Contig38	52,690,105	52,691,105	30,826,251	8
**EcWRKY-115**	Contig38	52,741,079	52,742,079	49,974	8
**EcWRKY-2**	Contig4	4,728,125	4,729,125	0	
**EcWRKY-98**	Contig4	13,843,136	13,844,136	9,114,011	
**EcWRKY-75**	Contig412	30,001	31,001	0	9
**EcWRKY-118**	Contig412	49,889	50,889	18,888	9
**EcWRKY-121**	Contig50	7,114,629	7,115,629	0	10
**EcWRKY-89**	Contig50	7,176,235	7,177,235	60,606	10
**EcWRKY-77**	Contig6	1097717	1098717	0	
**EcWRKY-129**	Contig6	8,795,083	8,796,083	7,696,366	
**EcWRKY-95**	Contig8	6,193,760	6,194,760	2,602,323	
**EcWRKY-37**	Contig8	15,066,849	15,067,849	8,872,089	
**EcWRKY-125**	Contig8	17,606,962	17,607,962	2,539,113	
**EcWRKY-92**	Contig8	18,288,170	18,289,170	680,208	
**EcWRKY-1**	Contig8	25,771,475	25,772,475	7,482,305	11
**EcWRKY-73**	Contig8	25,786,275	25,787,275	13,800	11
**EcWRKY-123**	Contig8	26,094,136	26,095,136	306,861	12
**EcWRKY-3**	Contig8	26,164,062	26,165,062	6,8926	12
**EcWRKY-53**	Contig884	302,823	303,823	0	13
**EcWRKY-90**	Contig884	31,8196	31,9196	14,373	13
**EcWRKY-20**	Scaffolds	5481496	5482496	0	
**EcWRKY-56**	Scaffolds	26,627,180	26,628,180	21,144,684	
**EcWRKY-97**	Scaffolds	35,583,751	35,584,751	8,955,571	
**EcWRKY-91**	Scaffolds	40,706,208	40,707,208	5,121,457	
**EcWRKY-43**	Scaffolds	49,356,212	49,357,212	8,649,004	
**EcWRKY-24**	Scaffolds	68,605,876	68606876	19,248,664	
**EcWRKY-78**	Scaffolds	68,822,451	68,823,451	215,575	
**EcWRKY-50**	Scaffolds	89,404,709	89,405,709	20,581,258	

Supplementary Table S12

The origins and ploidy levels of the cultivars and accessions of *E.curvula* analyzed by SSR.

**Accession/Name**	**Source**	**Ploidy**
**PI208214**	USDA	Diploid
**PI299919**	USDA	Diploid
**PI299920**	USDA	Diploid
**PI299928**	USDA	Diploid
**PI574506 (OTA)**	USDA	tetraploid
**PI234217 (Tanganyika)**	USDA	tetraploid
**Tanganyika**	INTA	tetraploid
**Don Walter**	INTA	tetraploid
**Don Pablo**	INTA	heptaploid
**Don Luis**	UNS-ACA	heptaploid

Supplementary Table S13
Parameters used to obtain the final *E. curvula* genome assembly with the Canu and FALCON software. When a parameter is not specified, it is because the default conditions were used.

**Version**	**Length_cutoff**	**Length_cutoff_pr**
Falconv5.4	5 kb	7,5 kb
Falconv5.3	5 kb	7,5kb
Falconv5.2	5 kb	7,5kb mincov5
Falconv5.1	5 kb	5kb mincov8
Falconv5.0	5 kb	5kb
Falconv4.5	7,5 kb	7,5kb mincov5
Falconv4.4	7,5 kb	7,5kb mincov5
Falconv4.3	7,5 kb	5kb micov5
Falconv4.1	7,5 kb	5kb
Falconv4.2	7,5 kb	7,5 kb
Falconv3.1	10 kb	5kb
Falconv3.2	10 kb	7,5kb
Falconv2.1	12 kb	5KB
Falconv2.2	12 kb	7,5kb
Falconv1.1	16,7 kb	1kb
Falconv1.2	16,7 kb	2,5kb
Falconv1.3	16,7 kb	5kb
Falconv1.4	16,7 kb	7.5kb
Falconv1.5	16,7 kb	10kb
**Version**	**ErrorRate**	**minOverlap**
Canuv1	ErrorRate 0,045	minOverlapLength=500
Canuv2	ErrorRate 0,045	minOverlapLength=1000
Canuv3	ErrorRate 0,01	minOverlapLength=500
CanuV4	ErrorRate 0,03	minOverlapLength=1000

Supplementary Table S14

Classification criteria used for *E. curvula* gene models. In the table the gene models included in each category are marked with an "X". The complete models are classified based on the presence of the start and stop codons and the homology includes matches with an e-value of 10-e10 and 90 % of coverage.

	HC1	HC2	LC1	LC2	LC3	TREP
Incomplete			X	X		
Complete	X	X				
Unimag	X					
Unipoa		X	X			
Trep						X
